# TET2 gene mutation status associated with poor prognosis of transition zone prostate cancer: a retrospective cohort study based on whole exome sequencing and machine learning models

**DOI:** 10.3389/fendo.2025.1568665

**Published:** 2025-04-14

**Authors:** Yutong Wang, Ailing Yu, Ziping Gao, Xiaoying Yuan, Xiaochen Du, Peng Shi, Haoyun Guan, Shuang Wen, Honglong Wang, Liang Wang, Bo Fan, Zhiyu Liu

**Affiliations:** 1 Department of Urology, Second Affiliated Hospital of Dalian Medical University, Dalian, Liaoning, China; 2 Liaoning Provincial Key Laboratory of Urological Digital Precision Diagnosis and Treatment, Second Affiliated Hospital of Dalian Medical University, Dalian, Liaoning, China; 3 Department of Urology, Liaoning Engineering Research Center of Integrated Precision Diagnosis and Treatment Technology for Urological Cancer, Dalian, Liaoning, China; 4 Dalian Key Laboratory of Prostate Cancer Research, Second Affiliated Hospital of Dalian Medical University, Dalian, Liaoning, China; 5 Department of Colorectal Surgery, Dalian Municipal Central Hospital, Dalian, Liaoning, China; 6 First Clinical College, Dalian Medical University, Dalian, Liaoning, China; 7 Department of Anatomy, College of Basic Medicine, Dalian Medical University, Dalian, Liaoning, China; 8 College of Humanities and Social Sciences, Dalian Medical University, Dalian, Liaoning, China; 9 Second Clinical College, Dalian Medical University, Dalian, Liaoning, China; 10 Department of Pathology, Dalian Friendship Hospital, Dalian, China

**Keywords:** transition zone, prostate cancer, whole-exome sequencing, driver genes, medication prediction, TET2 mutation, machine learning models

## Abstract

**Background:**

Prostate cancer (PCa) in the transition zone (TZ) is uncommon and often poses challenges for early diagnosis, but its genomic determinants and therapeutic vulnerabilities remain poorly characterized.

**Methods:**

Tumor mutational landscape was characterized in nine patients with TZ PCa, identifying somatic variants through whole-exome sequencing (WES). Novel candidate variants relevant to driver gene were selected using rare-variant burden analysis. Kaplan-Meier curves with log-rank testing and Cox regression models were applied to evaluate the prognostic significance of selected mutant driver gene and clinicopathological characteristics in a cohort of 132 patients with TZ PCa. Significant prognostic determinants were integrated into a validated nomogram for individualized prediction of 3-, 4-, and 5-year biochemical recurrence-free survival (BRFS) and overall survival (OS) probabilities. Eight machine learning algorithms were employed to develop BRFS and OS prediction models in a cohort.

**Results:**

A total of 5,036 somatic single nucleotide variants (SNVs) and 587 somatic insertion and deletion (INDELs) were discovered. Among eight driver gene mutations which were verified through Sanger sequencing, TET2 gene, with high mutation frequency and potential targeted drug relevance (bromodomain inhibitors and DOT1L inhibitors) was selected for further validation. Retrospective cohort study demonstrated that TET2 mutant status was significantly associated with Gleason score (p = 0.004) and distant metastasis (p = 0.002). Furthermore, TET2 mutant status was significantly correlated with inferior BRFS and OS and served as an independent predictor. Comparative evaluation of eight algorithms revealed the GBM model achieved superior discriminative ability for BRFS (AUC for 3-year: 0.752, 4-year: 0.786, 5-year: 0.796). The predictive model based on the GBM machine learning algorithm achieved the best predictive performance for OS (AUC for 3-year: 0.838, 4-year: 0.915, 5-year: 0.868). The constructed predictive nomogram provided evidence that TET2 mutant status integration conferred statistically significant improvements in model accuracy and clinical predictive value.

**Conclusion:**

Our study elucidated the distinct genetic basis of prostate cancer in the transition zone and identified TET2 mutation as an independent prognostic determinant in TZ PCa. However, the limited sample size of this study necessitates cautious interpretation of these findings, and further validation in larger cohorts is warranted to confirm their generalizability.

## 1 Introduction

Prostate cancer (PCa) represents the second most prevalent malignancy in male populations worldwide according to global incidence statistics, with nonmelanoma skin cancers being observed as the most prevalent malignancy in males (1, 2). Anatomical distribution patterns demonstrate that 20–25% of PCa cases originate from the transition zone (TZ), with significant epidemiological variations being observed across ethnic groups. Notably, Asian populations demonstrate disproportionately higher TZ PCa incidence rates compared to Western cohorts, thereby suggesting underlying ethnicity-specific molecular pathogenesis (3, 4). Current diagnostic protocols employ multiparametric magnetic resonance imaging (mpMRI) as the primary imaging modality for patients presenting with elevated prostate-specific antigen (PSA) levels, followed by histological confirmation via biopsy or postprostatectomy analysis (5). Although the use of mpMRI has improved the accuracy of TZ tumor detection compared with conventional methods (6–8), several critical diagnostic challenges persist. First, histological differentiation between TZ malignancies and concurrent benign prostatic hyperplasia (BPH) remains problematic due to architectural similarities in glandular proliferation patterns (9). Second, standard PSA-based screening protocols fail to identify a substantial proportion of TZ PCa cases with normal PSA values, thereby resulting in missed opportunities for MRI triage. Third, even among PSA-elevated patients, approximately 33% of TZ lesions evade mpMRI detection due to isointense signals overlapping with stromal hyperplasia components (7, 8, 10). The clinical implications of these diagnostic limitations are twofold. Although 70–80% of TZ PCa cases exhibit indolent biological behavior (Gleason score ≤6), histopathological analyses of radical prostatectomy specimens have revealed that 9.2% of index tumors contain Gleason grade 4/5 components. These aggressive subtypes demonstrate troublesome progression patterns, with 57% of the subtypes exhibiting prostatic capsular penetration (8). Given the persistent limitations in current diagnostic paradigms, including histological ambiguity, PSA screening inadequacy, and mpMRI interpretation challenges, the development of novel diagnostic technologies is imperative for increasing detection accuracy and enabling risk-stratified clinical management.

Although traditional diagnostic approaches have achieved performance plateaus, molecular diagnostics has emerged as a transformative paradigm in oncological practice, particularly for implementing precision medicine strategies (11). As a cornerstone technique of molecular profiling, whole-exome sequencing (WES) enables the systematic identification of protein-coding genomic alterations. In the context of transition zone prostate cancer, this technology offers several critical clinical advantages. First, mutational signature analysis can decipher the predominant somatic mutation patterns in TZ PCa, thus elucidating the etiological processes driving malignant transformation (12). Second, the identification of recurrent driver mutations can reveal the oncogenic mechanisms governing tumor proliferation and invasion, thereby providing molecular biomarkers to complement current histopathological diagnostics. Third, the computational prediction of targeted therapies and the anticipatory detection of resistance-conferring mutations can enable data-driven therapeutic optimization, thus potentially improving treatment response rates while minimizing systemic toxicity.

To address existing diagnostic gaps, we first reported nine cases of PCa occurring in the TZ and performed whole exome sequencing on these tumor tissues to explore genetic variants and tumor mutational signatures. Retrospective cohort study of 132 TZ PCa patients were included to further analyze the independent prognostic factors and the effect of TET2 on overall survival (OS) and biochemical recurrence-free survival (BRFS). Subsequently, driver mutations were discovered and validated by Sanger sequencing. Finally, targeted drug prediction and drug resistance mutation prediction analyses were performed to provide insights into the treatment of prostate cancer in the transition zone.

## 2 Materials and methods

### 2.1 Whole-exome sequencing analysis

#### 2.1.1 Tissue samples

The samples of nine patients with TZ PCa were obtained from the Second Affiliated Hospital of Dalian Medical University. The study was approved by the Ethics Committee of the Second Affiliated Hospital of Dalian Medical University. Written informed consent for the use of tissues and for the publication of this article was obtained from all of the patients who participated in the study. All of the study procedures conformed to the standards of the institutional research committees and were in accordance with the Helsinki Declaration guidelines.

#### 2.1.2 DNA extraction

DNA extraction was meticulously conducted following a standardized protocol. Paraffin-embedded samples were processed using the GeneRead DNA FFPE Tissue Kit (Qiagen, Hilden, Germany) following the manufacturer’s guidelines. DNA degradation and potential contamination were subsequently evaluated using 1% agarose gel electrophoresis. The genomic DNA concentration was assessed using a Qubit^®^ DNA assay kit and measured with a Qubit^®^ 2.0 fluorometer (Invitrogen, USA).

#### 2.1.3 Library preparation and sequencing

Genomic DNA was initially fragmented into segments of 180 to 280 base pairs via a Covaris instrument (Covaris, Massachusetts, USA). The Agilent SureSelect Human All Exon V6 Kit (Agilent Technologies, CA, USA) was utilized to capture and enrich the entire exome, followed by purification using the AMPure XP system (Beckman Coulter, Beverly, MA, USA). The quality and concentration of the libraries were evaluated using quantitative polymerase chain reaction (qPCR), an Agilent Bioanalyzer 2100 and a Qubit 2.0 Fluorometer (Invitrogen, USA). Afterwards, sequencing of the libraries was performed on an Illumina HiSeq platform (Illumina, San Diego, CA, USA) with 150 bp paired-end reads, thereby achieving average sequencing depths of >100× and >95% target coverage at ≥10×.

#### 2.1.4 Quality control

The raw data generated from the HiSeq platform were documented in the FASTQ format. To facilitate subsequent bioinformatics analyses, it was imperative to discard undesirable elements within the data, such as unidentified nucleotides, low-quality nucleotides and adapter contaminants. A set of criteria was established to filter out paired reads that did not meet the desired quality standards. The following types of reads were excluded: (i) reads containing adapter contamination, (ii) reads containing more than 10% uncertain bases and (iii) reads containing more than 50% low-quality bases.

#### 2.1.5 Bioinformatics analysis

The sequencing reads were aligned to the reference genome (B37) via the BWA-MEM algorithm (v0.1.22) (13). SAMtools (v1.0) was used to sort the generated files in binary alignment map (BAM) format. Sambamba (v0.4.7) was used to identify and mark duplicate reads (14). Base quality score recalibration (BQSR) and local realignment around insertion and deletions (INDELs) were performed following GATK Best Practices (15). Typically, to obtain more reliable single nucleotide polymorphisms (SNPs), the mapping rate of sequencing reads in human samples should be greater than 95%, and the sequencing depth should be greater than 10X.

#### 2.1.6 Variant calling, indel detection and somatic mutation calling

SNPs and INDELs were identified and filtered using SAMtools (v1.0) and BCFtools. Germline mutations were first detected using SAMtools, and somatic single nucleotide variants (SNVs) were subsequently identified through a filtering process based on germline mutation results. Specifically, somatic SNVs detection was performed by screening germline variants under the following criteria:

1. Variants with an allele-specific allele fraction (AsAF) >0.95 were retained;
2. Variants with allele frequencies <0.1 in the 1000 Genomes (1000g), ExAC_ALL, ExAC_EAS, and NOVOdb databases were retained;
3. Variants located in functionally annotated exonic regions were retained.

Variant call formats (VCFs) generated in earlier phases were annotated using ANNOVAR (2013Aug23), which integrates the dbSNP, 1000 Genomes databases, and other relevant resources (16, 17). Mutation structures (e.g., mRNAs, noncoding RNAs, small RNAs, microRNAs) were annotated using the NCBI RefSeq and Gencode databases. Somatic INDELs were detected using Strelka (v2.9.10) with default parameters (18, 19).

#### 2.1.7 Mutational spectrum and mutational signature analysis

Initially, the somatic SNVs in each sample were divided into six substitutions (C>A, C>G, C>T, T>A, T>C, and T>G) as the mutational spectrum. Somatic SNVs were expanded into 96 different contexts (described as mutational signatures) by considering the location of the base at 1 bp upstream and downstream. These mutation contexts were then decomposed into three signatures using the nonnegative matrix factorization (NMF v0.22) method. Furthermore, the signatures were compared with known mutational signatures in the Catalogue of Somatic Mutations in Cancer (COSMIC) databases to identify specific mutational processes or biological factors (20).

#### 2.1.8 Identification of potential driver mutations and significantly mutated genes

Mutations in nine tumor samples were compared to known driver mutations via in-house software in the Cancer Gene Census (CGC) (http://cancer/sanger.ac.uk/cancergenome/projects/census), Bert Vogelstein125, SMG127, and Comprehensive435 databases to identify potential driver mutations for TZ PCa. The Mutational Significance in Cancer (MuSiC) (Genome-Model-Tools-Music-0.04) tool was used to detect significantly mutated genes (SMGs) in nine samples, which comprehensively included somatic SNVs, INDELs and other mutations (21). In this context, significantly mutated genes are defined as those genes exhibiting mutation rates that are significantly greater than the background mutation rate (BMR).

#### 2.1.9 Sanger sequencing

The driver genes were validated via Sanger sequencing. Sanger sequencing was performed through optimized experimental procedures beginning with DNA extraction using proprietary kits that were selected based on sample types, with DNA quality being assessed by using 1% agarose gel electrophoresis (120–180 V in 1X TAE buffer) and spectrophotometric verification (OD260/280 ratio: 1.7–2.0). Target-specific primers (18–30 bp) were designed using Primer Premier 5 with the following parameters: Tm, 55–65°C; GC content, 40–70%; and strict avoidance of secondary structures or 3’ GC-rich regions. PCR amplification was performed in 25 μl reactions containing 1 μl of template DNA, 0.2 U of Taq polymerase, 200 μM of dNTPs, and 1 μM of each primer via thermal cycling conditions of initial denaturation (95°C/5 min), followed by 10 touchdown cycles (94°C/30 s, 63–58°C/30 s, and 72°C/30 s) and 30 standard cycles (95°C/30 s, 58°C/30 s, and 72°C/30 s), along with a final extension (72°C/10 min). Amplification success was confirmed via 1% agarose gel electrophoresis (150 V, 20 min) by using Thermo SM0331 and Sangon B500347 DNA markers, followed by gel purification of the target bands (150–300 bp) using SanPrep spin columns. Bidirectional sequencing reactions were analyzed on ABI platforms with data processing conducted via Chromas and SeqMan software for base calling and sequence alignment, with rigorous quality control being maintained via peak resolution evaluation and bidirectional confirmation of ambiguous positions.

#### 2.1.10 Identification of targeted drug mutations and drug resistance mutations

Significantly mutated genes in tumour samples were compared with those in the NovoDrug database to screen for mutations associated with existing targeted drugs for targeted drug prediction. The NovoDrug database integrates established targeted drug databases such as PharmGKB, My Cancer Genome, and KEGG, as well as incorporating clinical trial results from sources such as ClinicalTrials.gov. Comparisons between somatic mutations in tumour samples and the NovoDR Drug Resistance Gene database were conducted to identify potential cancer drug-resistant mutations, which is important for guiding clinical medication decisions and assessing patient prognosis. The NovoDR Drug Resistance Gene database consolidates data from CGC, Bert Vogelstein, SMG127, and other comprehensive repositories. Currently, experimental findings from over 100 tumour resistance studies (encompassing 91 drug resistance genes, 95 anticancer agents, and 35 cancer types) are aggregated. These data span multiple resistance mechanisms, including drug transport, drug metabolism, alterations in drug targets, enhanced DNA mismatch repair capacity, and antiapoptotic signaling pathways. The analysis of targeted drug prediction and drug-resistant mutation screening was performed using in-house scripts (v0.22). Mutation sites with sequencing depths of less than 8× and mutation sites located in the intergene, noncoding and intron regions as well as the mutation sites of synonymous mutations were filtered out.

### 2.2 Retrospective cohort study

#### 2.2.1 Retrospective cohort patients data

Following the exclusion of individuals with secondary malignancies, we analyzed clinical records of 132 patients with pathologically confirmed TZ PCa who were treated at our institution from 2017 to 2024. Ethical approval was obtained from the Institutional Research Ethics Committees, with written informed consent acquired from all participants or legal guardians. This investigation adhered to the Declaration of Helsinki and relevant regulatory standards. Collected clinicopathological parameters included: age in years at surgery, preoperative PSA levels, prostate weight, Gleason score, pathological T stage, lymph node metastasis, distant metastasis, seminal vesicle invasion, perineural invasion.

#### 2.2.2 Statistical analysis

Baseline characteristics were compared between groups using χ² tests or Fisher’s exact tests as appropriate. Associations between clinicopathological parameters and TET2 mutation were evaluated through Pearson correlation analysis. Cox proportional hazards regression models were employed for both univariate and multivariate analyses to identify independent prognostic factors, with results expressed as hazard ratios (HRs) and 95% confidence intervals (CIs). Significant predictors from multivariable analysis (p<0.05) were subsequently incorporated into nomogram construction for visual prediction of survival outcomes. Survival outcomes including OS and BRFS were analyzed using Kaplan-Meier curves with log-rank testing for group comparisons. For predictive modeling, eight machine-learning algorithms were systematically evaluated: Lasso Cox, random survival forest (RSF), CoxBoost, generalized boosted regression modeling (GBM), support vector machine (Survival-SVM), eXtreme Gradient Boosting (XGBoost), supervised principal components (SuperPC), and partial least squares regression for Cox (plsRcox). Model performance was rigorously assessed through two complementary approaches: 1) time-dependent Receiver Operating Characteristic (ROC) analysis with Area Under the Curve (AUC) calculations, and 2) decision curve analysis (DCA) to evaluate clinical utility across various risk thresholds. Statistical analyses were performed using SPSS 13.0 (SPSS Inc.) and R software (R Foundation).

## 3 Results

### 3.1 Whole-exome sequencing

#### 3.1.1 Identification of SNPs and INDELs

**Supplementary Table S1** presents the clinical features of the nine patients. Whole-exome sequencing was performed on all of the tumour samples. A total of 802,278 SNPs and 106,175 INDELs were discovered in nine samples, with 11,077 novel SNPs and 14,397 novel INDELs observed in the genome (**Supplementary Table S2–S5**). In the entire genome, the transition/transversion ratio (TS: TV) was typically observed at approximately 2.2, whereas it was approximately 3.2 in the coding region, which demonstrated the precision of the SNP data. The TS: TV values of the nine samples in the genome are shown in **Supplementary Table S3**. **Supplementary Figures S1**, **S2** show the numbers of SNPs and INDELs, respectively, in various genomic and coding regions.

#### 3.1.2 Identification of somatic SNVs and INDELs

Somatic mutations, which are neither inherited from parents nor passed on to offspring, lead to the development of mutations that drive the growth and progression of tumors. A total of 5,036 somatic SNVs, which were mostly missense mutations, were detected (**Supplementary Table S6**). A total of 587 somatic INDELs, which were primarily nonframeshift mutations, were discovered (**Supplementary Table S7**). Although the number of frameshift mutations was less than the number of nonframeshift mutations in all of the tumour specimens, frameshift mutations were more likely to induce a shift in the entire reading frame due to the lengths of the inserted or missing bases (a noninteger multiple of three in the frameshift variations).

#### 3.1.3 Identification of the mutational spectrum and mutational signature

**Figure 1:**
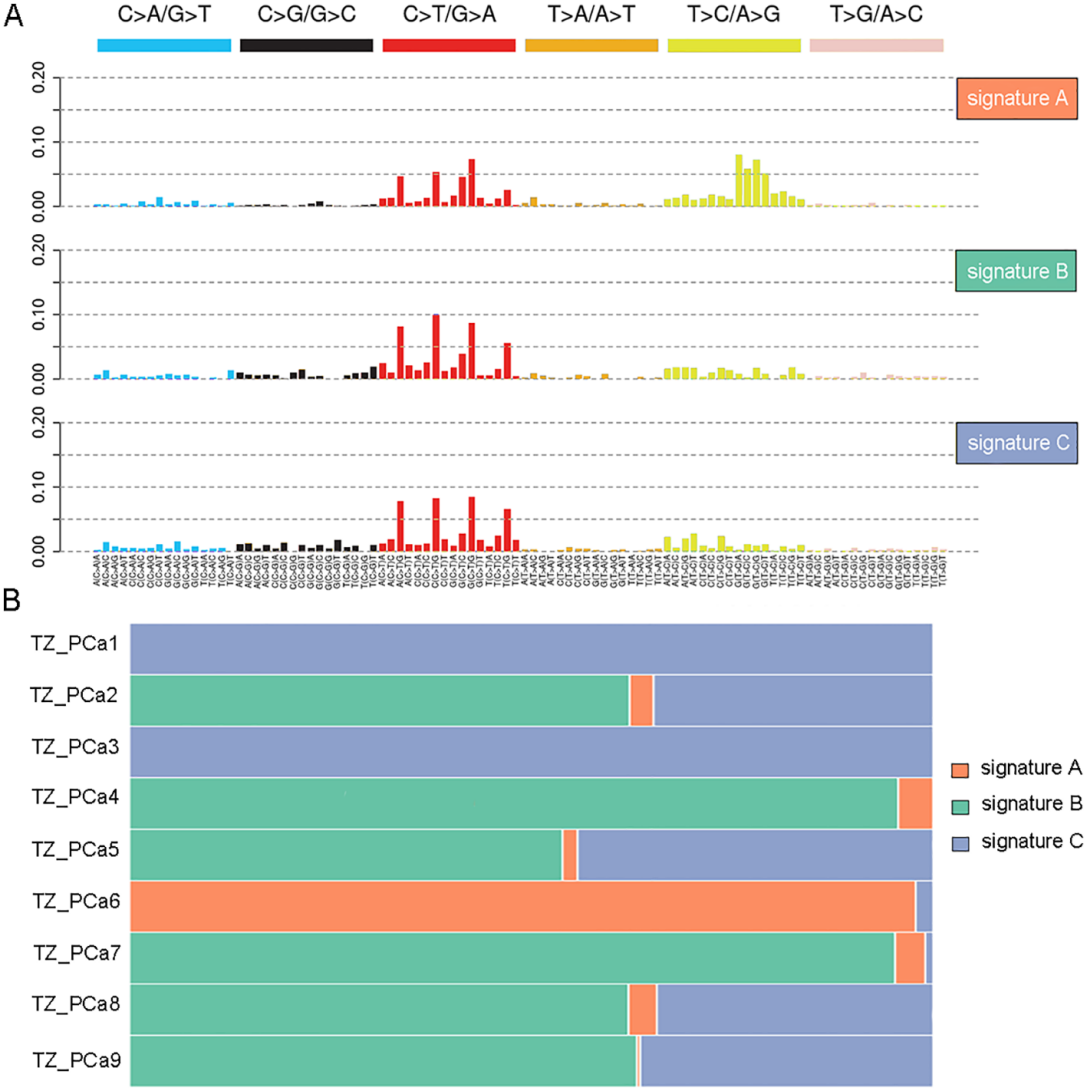
Mutation signature analysis in nine TZ PCa. Three signatures are recognized in nine TZ PCa using nonnegative matrix factorization (NMF) **(A)**, and the different proportions of three signatures in nine samples are protracted **(B)**.

Initially, the mutational spectrum revealed that prostate tumors occurring in the transition zone in different patients exhibited a similar pattern of single-base substitutions (SBSs), and the C>T/G>A transition was the most prevalent substitution in the samples (**Supplementary Figure S3**). Furthermore, an unsupervised analysis of the mutational signatures was conducted using the NMF method, and the SBSs were divided into signature A, signature B and signature C groups (**Figure 1A**). As a result, a high prevalence of signature A was detected in TZ_PCa6 sample, thus indicating a significant C>T/G>A mutation and T>C/A>G mutation; moreover, signature B and signature C were also detected in the other samples, thereby indicating a notable C>T/G>A mutation (**Figure 1B**). Finally, the mutational signatures of our samples were clustered with known mutational signatures in the COSMIC database (**Supplementary Figure S4A**). The cosine similarities were calculated and displayed in the heatmap, which demonstrated that signature A was similar to signature 21. Signatures B and C resembled each other and were also similar to signature 1 (**Supplementary Figure S4B**, **Supplementary Table S8**).

#### 3.1.4 Identification of driver mutations and significantly mutated genes

**Figure 2:**
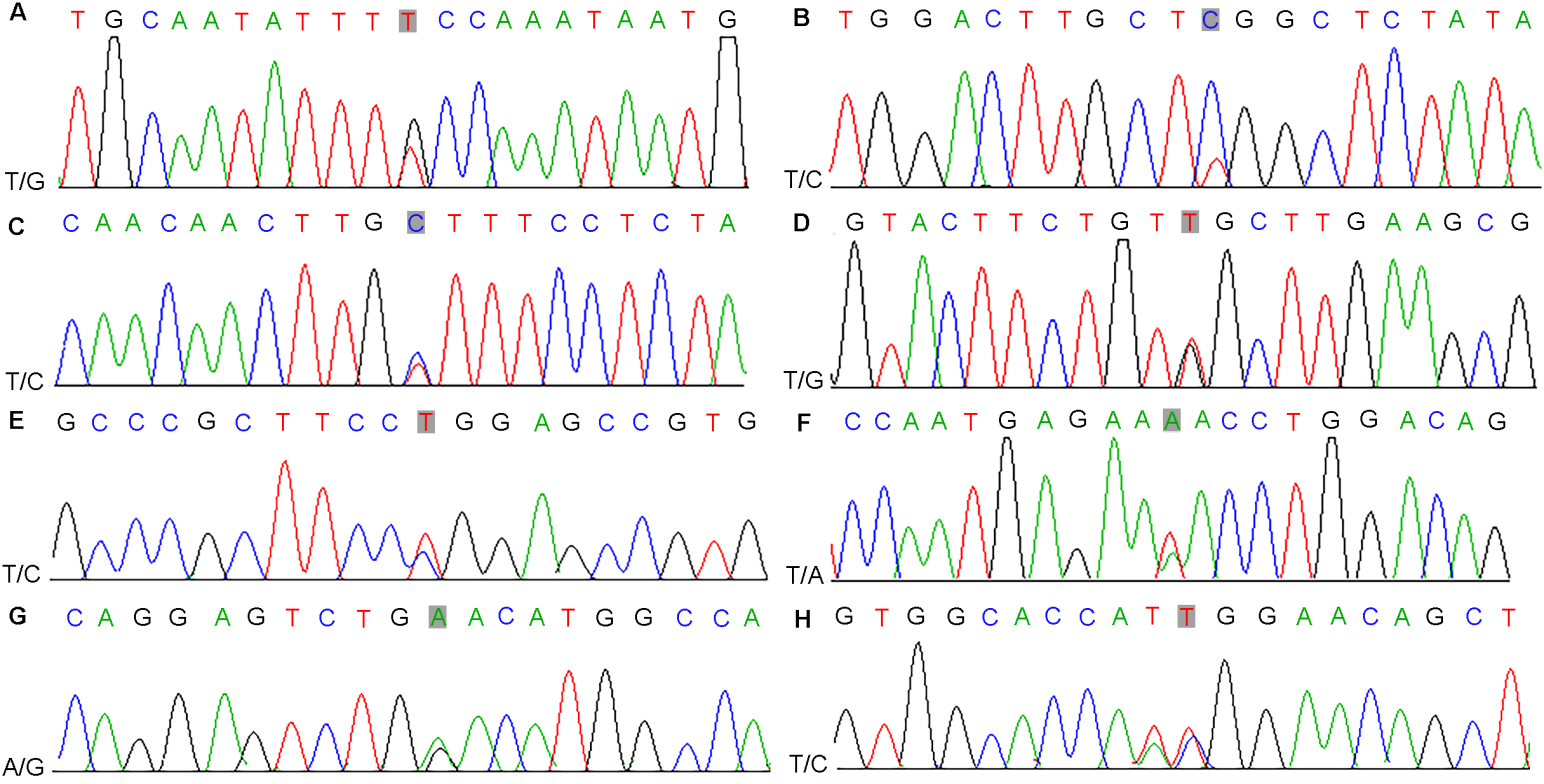
Results of nucleotide Sanger sequencing analysis of eight driver gene mutations (TET2, CDK12, CEP89, NKX2-1, ASPSCR1, ARID2, SPEG, and HOXC11) in nine samples **(A-H)**.

Driver genes play critical roles in the proliferation and spread of tumors and confer a selective growth advantage. Eight genes (TET2, CDK12, CEP89, NKX2-1, ASPSCR1, ARID2, SPEG, and HOXC11) with driver mutations were identified in our samples and verified via Sanger sequencing (**Table 1**, **Figure 2**; **Supplementary Figure S5**). Significantly mutated genes in the nine samples are shown in a heatmap that comprehensively includes somatic SNVs, INDELs and other mutations (**Supplementary Figure S6**).

**Table 1:** Analysis results of driver mutations in TZ prostate cancers.

Gene Symbol	Chromosome	Position	Reference allele	Alternative allele	Variant classification	Amino acid change
TET2	4	106157703	T	G	Missense Mutation	NM_001127208:exon3:c.T2604G:p.F868L |NM_017628:exon3:c.T2604G:p.F868L
CDK12	17	37665992	C	T	Missense Mutation	NM_015083:exon7:c.C2644T:p.R882W |NM_016507:exon7:c.C2644T:p.R882W
CEP89	19	33409204	T	C	Missense Mutation	NM_032816:exon13:c.A1310G:p.N437S
NKX2-1	14	36987054	T	G	Missense Mutation	NM_003317:exon2:c.A545C:p.Q182P |NM_001079668:exon3:c.A635C:p.Q212P
ASPSCR1	17	79941518	C	T	Missense Mutation	NM_001251888:exon3:c.C247T:p.R83W |NM_024083:exon3:c.C247T:p.R83W
ARID2	12	46254690	A	T	Missense Mutation	NM_152641:exon16:c.A4880T:p.K1627I
SPEG	2	220337041	G	A	Missense Mutation	NM_005876:exon15:c.G3928A:p.D1310N
HOXC11	12	54367281	C	T	Missense Mutation	NM_014212:exon1:c.C256T:p.R86W

#### 3.1.5 Analysis of targeted drug prediction and drug resistance mutation prediction

**Figure 3:**
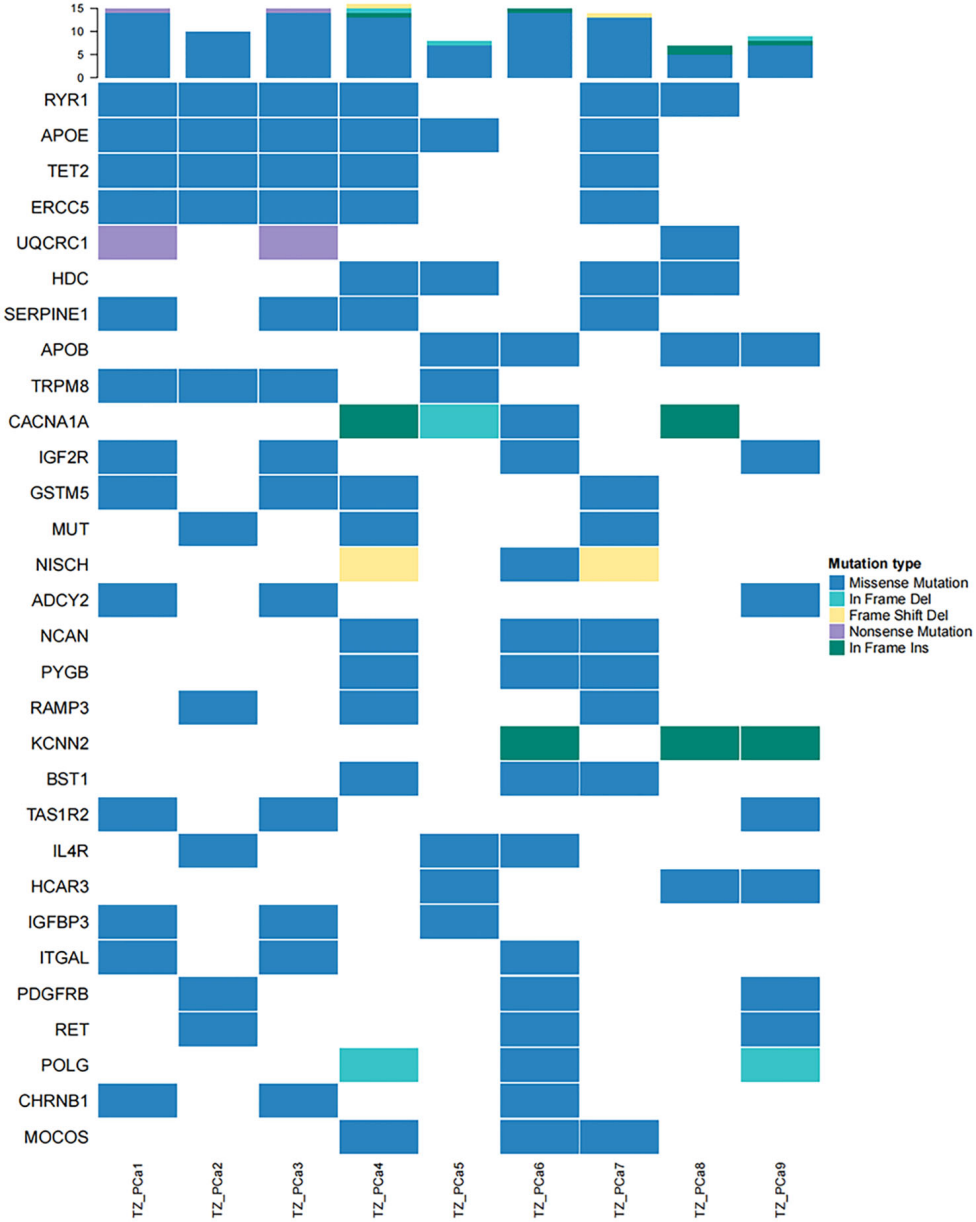
High-frequency mutations in genes affected by existing targeted drugs are presented in a heatmap. TET2 mutations were identified in TZ_PCa1-4 and TZ_PCa7.

Compared with conventional therapies, cancer-targeted therapy is associated with more accurate targeting of specific mutation sites in tumour cells, a reduction in drug side effects and an increase in drug efficacy. Significantly mutated genes, which are classified into oncogenes and tumour suppressor genes, typically serve as potential therapeutic targets. This approach represents a cornerstone of precision medicine in cancer treatment. The SMGs in the samples were compared to those in the NovoDrug database, which contains consolidated data from the PharmGKB database, My Cancer Genome database, KEGG database and other targeted drug databases, to screen SMGs of existing targeted drugs (**Figure 3**). The drug name and drug type were determined to guide the clinical treatment (**Supplementary Table S9**). Notably, two types of potential targeted drugs (bromodomain inhibitors and DOT1L inhibitors) were discovered for the identified driver mutation site within TET2 in our study. Drug resistance mutations are the main cause of chemotherapy failure and often lead to tumour recurrence and metastasis. Somatic mutations in genes related to drug resistance in PCa of the TZ are shown in **Supplementary Table S10**. PCa occurring in the TZ (which harbors mutations such as an in-frame insertion at X:66765174 or a missense mutation at X:66931247 in the AR gene) may exhibit resistance to drugs such as leuprolide and bicalutamide, which are commonly used to treat PCa.

### 3.2 Retrospective cohort

#### 3.2.1 Baseline characteristics

The baseline clinicopathological characteristics of the 132 patients with TZ PCa stratified by TET2 mutation status are summarized in **Table 2**. The cohort had a median age of 74 years at diagnosis (range from January 2017 to December 2024), with comparable age distributions observed between the TET2 mutant (n=109, 82.6%) and wild-type subgroups (n=23, 17.4%) (≤74 years: 51 [46.8%] vs. 10 [43.5%]; >74 years: 58 [53.2%] vs. 13 [56.5%]; p=0.772). Preoperative PSA levels and prostate weight showed no significant intergroup differences: 72.5% (79/109) of wild-type and 56.5% (13/23) of mutant patients presented with PSA ≤10 ng/mL (p=0.292), while prostate weight ≤46 g was observed in 53.2% (58/109) of wild-type versus 39.1% (9/23) of mutant cases (p=0.220). Notably, TET2 mutation exhibited significantly more aggressive pathological features. High-grade Gleason scores (≥4 + 3) were markedly enriched in the TET2 mutant subgroup (34.8% [8/23] vs. 9.2% [10/109] in wild-type, p=0.004), whereas low-grade disease (≤3 + 3) predominated in wild-type patients (72.5% [79/109] vs. 47.8% [11/23]). A trend toward advanced pathological T stage (≥T2) was observed in TET2 mutant cases (30.4% [7/23] vs. 13.8% [15/109] wild-type, p=0.051). A similar trend toward advanced pathological T stage (≥T2) was observed in TET2 mutant patients, though this difference approached but did not reach statistical significance (14.7% vs. 30.4%, p=0.051). Metastatic events were strongly associated with TET2 mutational status. Lymph node involvement occurred exclusively in mutant patients (8.7% [2/23] vs. 0.9% [1/109] wild-type, p=0.023), and distant metastases were detected only in the mutant cohort (8.7% [2/23] vs. 0% wild-type, p=0.002). Seminal vesicle invasion (wild-type: 4.6% [5/109]; mutant: 0%) and perineural invasion rates (wild-type: 6.4% [7/109]; mutant: 8.7% [2/23]) did not differ significantly between groups (both p>0.05).

**Table 2:** Baseline characteristics of patients with prostate transition zone tumours.

Variable	TET2 non-mutant	TET2 Mutant	p-value
Age in years at surgery			0.772
≤74	51	10	
>74	58	13	
Preoperative PSA level, ng/mL			0.292
≤10	79	13	
10-20	13	5	
>20	17	5	
Prostate weight (g)			0.220
≤46	58	9	
>46	51	14	
Gleason score			0.004
≤3+3	79	11	
3+4	20	4	
≥4+3	10	8	
Pathological T stage			0.051
T1c	94	16	
T2 or more	15	7	
Lymph node metastasis			0.023
Negative	108	21	
Positive	1	2	
Distant metastasis			0.002
Negative	109	21	
Positive	0	2	
Seminal vesicle invasion			0.295
Negative	104	23	
Positive	5	0	
Perineural invasion			0.694
Negative	102	21	
Positive	7	2	

#### 3.2.2 Independent prognostic factors and nomogram for OS

**Figure 4:**
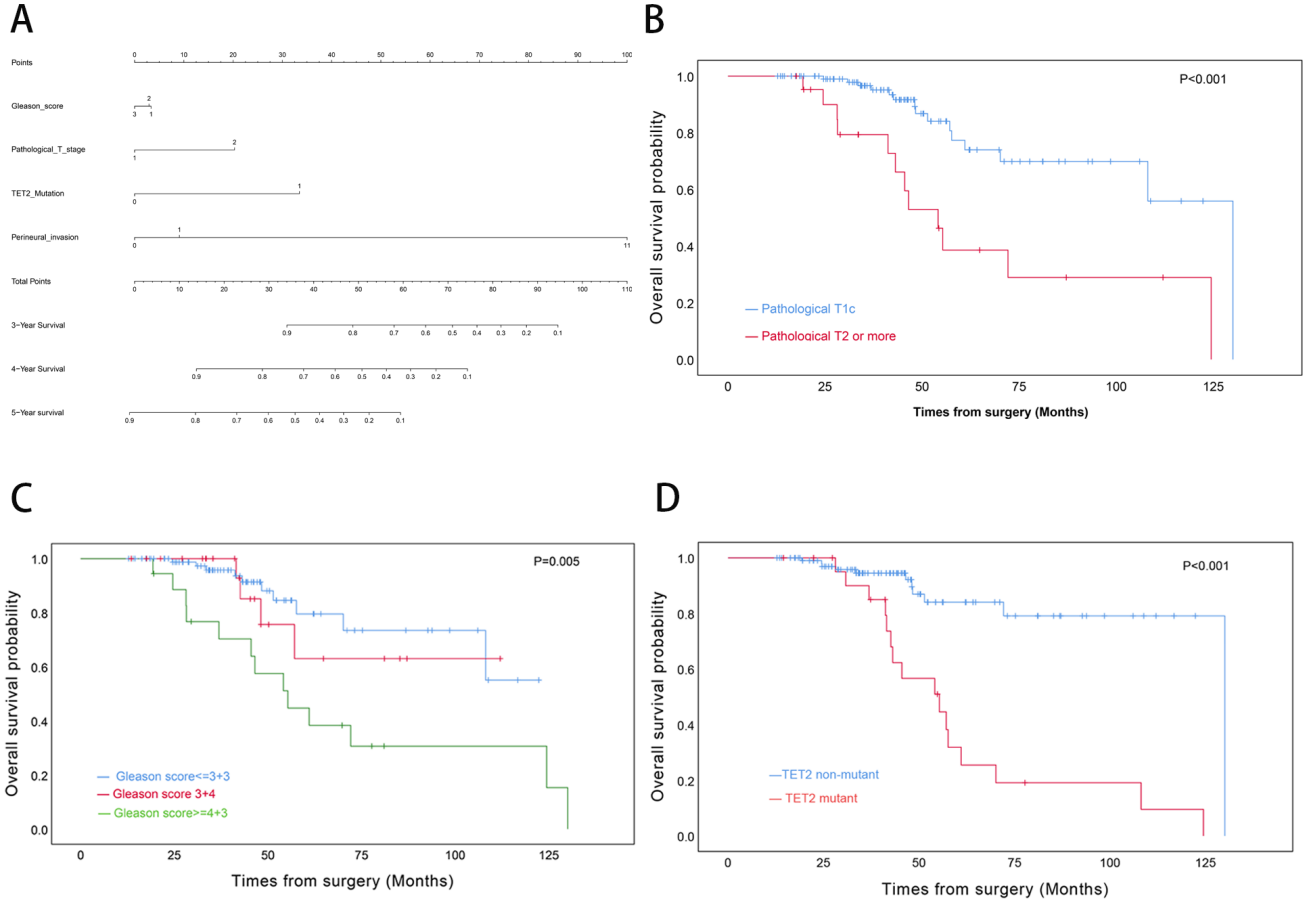
Prognostic Modeling and Survival Analysis in Transition Zone Prostate Cancer. **(A)** Nomogram integrating clinicopathological variables for predicting 3-, 4-, and 5-year overall survival probabilities. **(B)** Kaplan-Meier curves stratified by pathological T stage. **(C)** Survival differentiation across Gleason grade groups. **(D)** Comparative survival analysis by TET2 mutation status.

For OS, univariate Cox regression analysis revealed significant associations with Gleason score (≥4 + 3 vs ≤3 + 3: HR=3.70, p=0.003), pathological T stage (T2–4 vs T1c: HR=3.83, p<.0001), and TET2 mutation status (HR=5.95, p<0.001). However, only TET2 mutation retained independent prognostic significance in multivariate analysis (adjusted HR=4.58, p=0.001) (**Table 3**). A novel nomogram incorporating Gleason score stratification, pathological T classification, TET2 mutation, and perineural invasion status was developed for individualized OS estimation (**Figure 4A**). The model demonstrated stable discriminative performance across follow-up intervals, with time-specific AUC values reaching 0.700 (3-year), 0.779 (4-year), and 0.702 (5-year). Kaplan-Meier analysis delineated three significant predictors of inferior OS in TZ PCa: advanced pathological T stage (T2-4 vs T1c, p<0.001), elevated Gleason score (≥4 + 3 vs ≤3 + 3, p=0.005), and TET2-mutant status (mutant vs non mutant, p<0.001) (**Figures 4B-D**).

**Table 3:** Univariate and multivariate analyses of risk factors associated with OS.

Overall Survival	Univariate analysis		Multivariate analysis	
	HR (95%CI)	P value	HR (95%CI)	P value
Age				
>74 vs ≤74	0.96 (0.43-2.16)	0.928		
Preoperative PSA level				
10-20 vs ≤10	0.91(0.30-2.71)	0.862		
>20 vs ≤10	0.73 (0.24-2.20)	0.581		
Prostate weight				
>46 vs ≤46	1.30 (0.58-2.92)	0.526		
Gleason score				
3+4 vs ≤3+3	1.38 (0.43-4.39)	0.590	1.11 (0.34-3.68)	0.861
≥4+3 vs ≤3+3	3.70 (1.56-8.80)	0.003	1.22 (0.41-3.61)	0.719
Pathological T stage				
T_2_ or more vs T_1c_	3.83 (1.75-8.35)	<0.001	2.37 (0.92-6.06)	0.073
Lymph node metastasis				
N_1_ vs N_0_	0.84 (0.11-6.24)	0.863		
Distant metastasis				
M_1_ vs M_0_	1.87 (0.37-9.45)	0.450		
Seminal_vesicle_invasion				
Positive vs negative	0.80 (0.18-3.62)	0.777		
Perineural invasion				
Positive vs negative	1.14 (0.27-4.88)	0.860		
TET2 Mutation status				
Mutant vs non-mutant	5.95 (2.68-13.21)	<0.001	4.58 (1.86-11.27)	0.001

OS, overall survival; HR, hazard ratio; CI, confidence interval.

#### 3.2.3 Feature selection and model comparisons for OS

**Figure 5:**
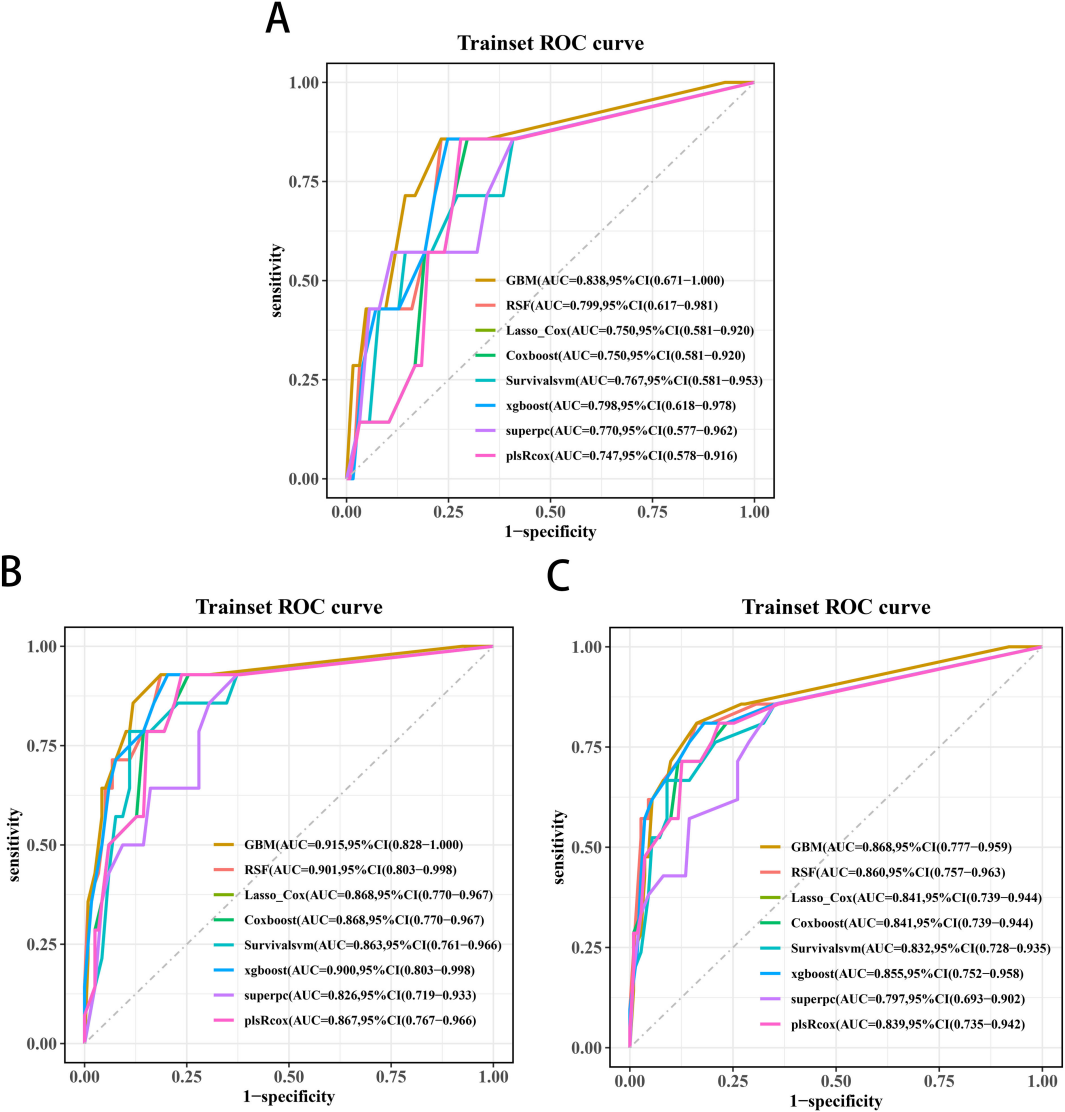
Receiver operating characteristic curves of the 3-year **(A)**, 4-year **(B)**, and 5-year OS **(C)** prediction models for transition zone prostate cancer.

**Figure 6:**
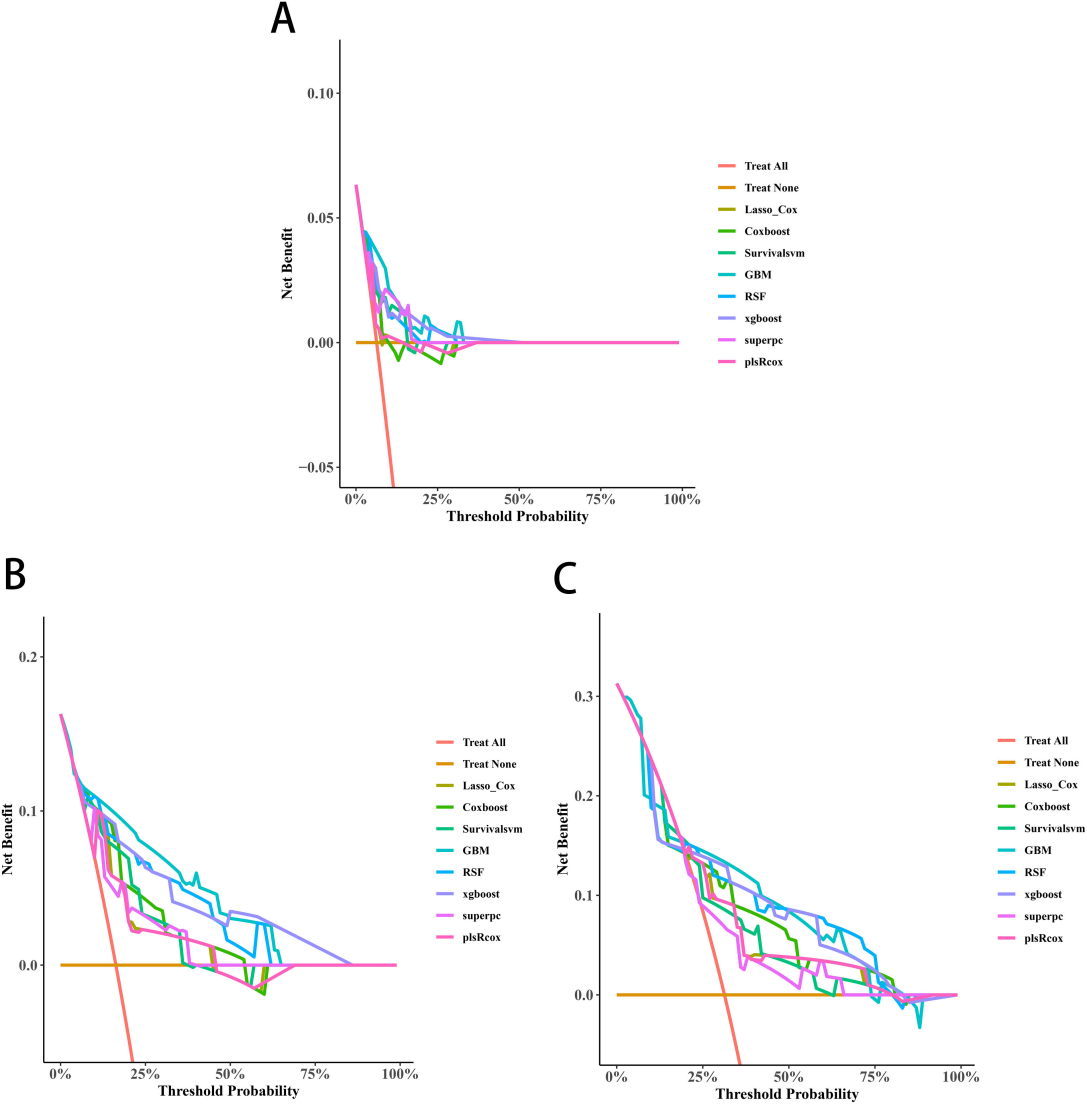
Decision curve analyses (DCAs) curves of the 3-year **(A)**, 4-year **(B)**, and 5-year OS **(C)** prediction models for transition zone prostate cancer.

Univariate Cox screening selected three statistically significant features (Gleason score, pathological T stage and TET2 mutation status) for predictive model construction. Comparative evaluation of eight algorithms revealed the GBM model achieved superior discriminative ability across all timepoints: 3-year (AUC=0.838, 95%CI:0.671-1.000; **Figure 5A**), 4-year (AUC=0.915, 95%CI:0.828-1.000; **Figure 5B**), and 5-year OS prediction (AUC=0.868, 95%CI:0.777-0.959; **Figure 5C**). DCA further confirmed the GBM model’s clinical decision-making utility (**Figures 6A-C**).

#### 3.2.4 Independent prognostic factors and nomogram for BRFS

**Figure 7:**
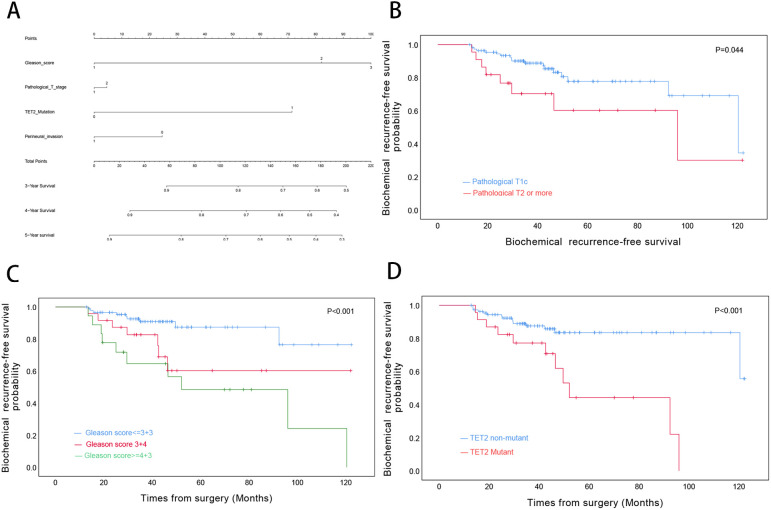
Biochemical Recurrence Risk Stratification in Transition Zone Prostate Cancer. **(A)** Predictive nomogram for 3-, 4-, and 5-year biochemical recurrence-free survival probabilities. **(B)** BRFS analysis by pathological T stage. **(C)** BRFS differentiation across Gleason grade categories. **(D)** Comparative BRFS analysis stratified by TET2 mutational status.

BRFS analysis demonstrated differential prognostic impacts across variables. Both univariate and multivariate models identified Gleason score (3 + 4 vs ≤3 + 3: adjusted HR=2.83, p=0.045; ≥4 + 3 vs ≤3 + 3: adjusted HR=3.48, p=0.021 and TET2 mutation (adjusted HR=2.59, p=0.033) as consistent independent predictors. While pathological T stage showed univariate significance (T2–4 vs T1c: HR=2.32, p=0.050), this association was attenuated in multivariate adjustment (p=0.921) (**Table 4**). A novel nomogram model was developed to predict the 3-, 4-, and 5-year OS rates for individual TZ PCa patients (**Figure 7A**). Integrating four critical prognostic factors: Gleason score, pathological T stage, TET2 mutation status, and perineural invasion, the nomogram exhibits a gradual enhancement in predictive precision over time, with time-dependent ROC-AUC values of 0.676, 0.725, and 0.734 for predicting 3-, 4-, and 5-year OS, respectively. Kaplan-Meier survival curve identified three clinicopathological parameters significantly associated with biochemical recurrence risk: pathological T stage (T2-4 vs T1c, p=0.044), Gleason score (p<0.001), and TET2 variants (mutant vs non mutant, p<0.001) (**Figures 7B-D**).

**Table 4:** Univariate and multivariate analyses of risk factors associated with BRFS.

Biochemical Recurrence-free Survival	Univariate analysis		Multivariate analysis	
	HR (95%CI)	P value	HR (95%CI)	P value
Age				
>74 vs ≤74	2.00 (0.84-4.78)	0.119		
Preoperative PSA level				
10-20 vs ≤10	1.34 (0.44-4.11)	0.608		
>20 vs ≤10	2.06 (0.86-4.96)	0.107		
Prostate weight				
>46 vs ≤46	1.10 (0.50-2.43)	0.807		
Gleason score				
3+4 vs ≤3+3	2.96 (1.10-7.97)	0.032	2.83 (1.02-7.83)	0.045
≥4+3 vs ≤3+3	5.24 (2.11-13.03)	<0.001	3.48 (1.21-10.04)	0.021
Pathological T stage				
T_2_ or more vs T_1c_	2.32 (1.00-5.37)	0.050	1.05 (0.41-2.66)	0.921
Lymph node metastasis				
N_1_ vs N_0_	1.26 (0.17-9.39)	0.824		
Distant metastasis				
M_1_ vs M_0_	3.89 (0.87-17.46)	0.076		
Seminal_vesicle_invasion				
Positive vs negative	2.22 (0.76-6.48)	0.146		
Perineural invasion				
Positive vs negative	1.04 (0.24-4.53)	0.963		
TET2 Mutation status				
Mutant vs non-mutant	3.76 (1.71-8.29)	0.001	2.59 (1.08-6.20)	0.033

BRFS, biochemical recurrence-free survival; HR, hazard ratio; CI, confidence interval.

#### 3.2.5 Feature selection and model comparisons for BRFS

**Figure 8:**
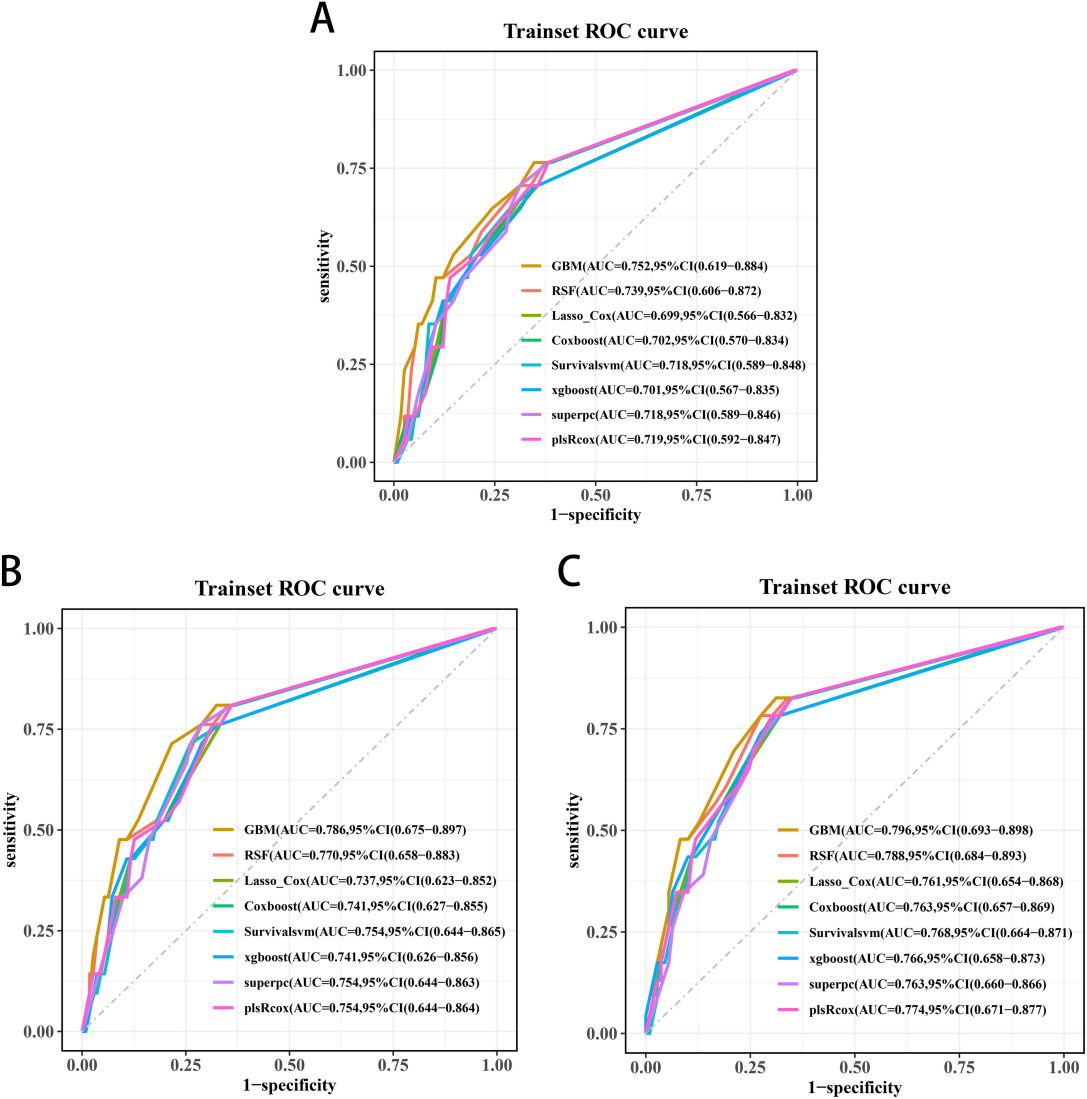
Receiver operating characteristic curves of the 3-year **(A)**, 4-year **(B)**, and 5-year BRFS **(C)** prediction models for transition zone prostate cancer.

**Figure 9:**
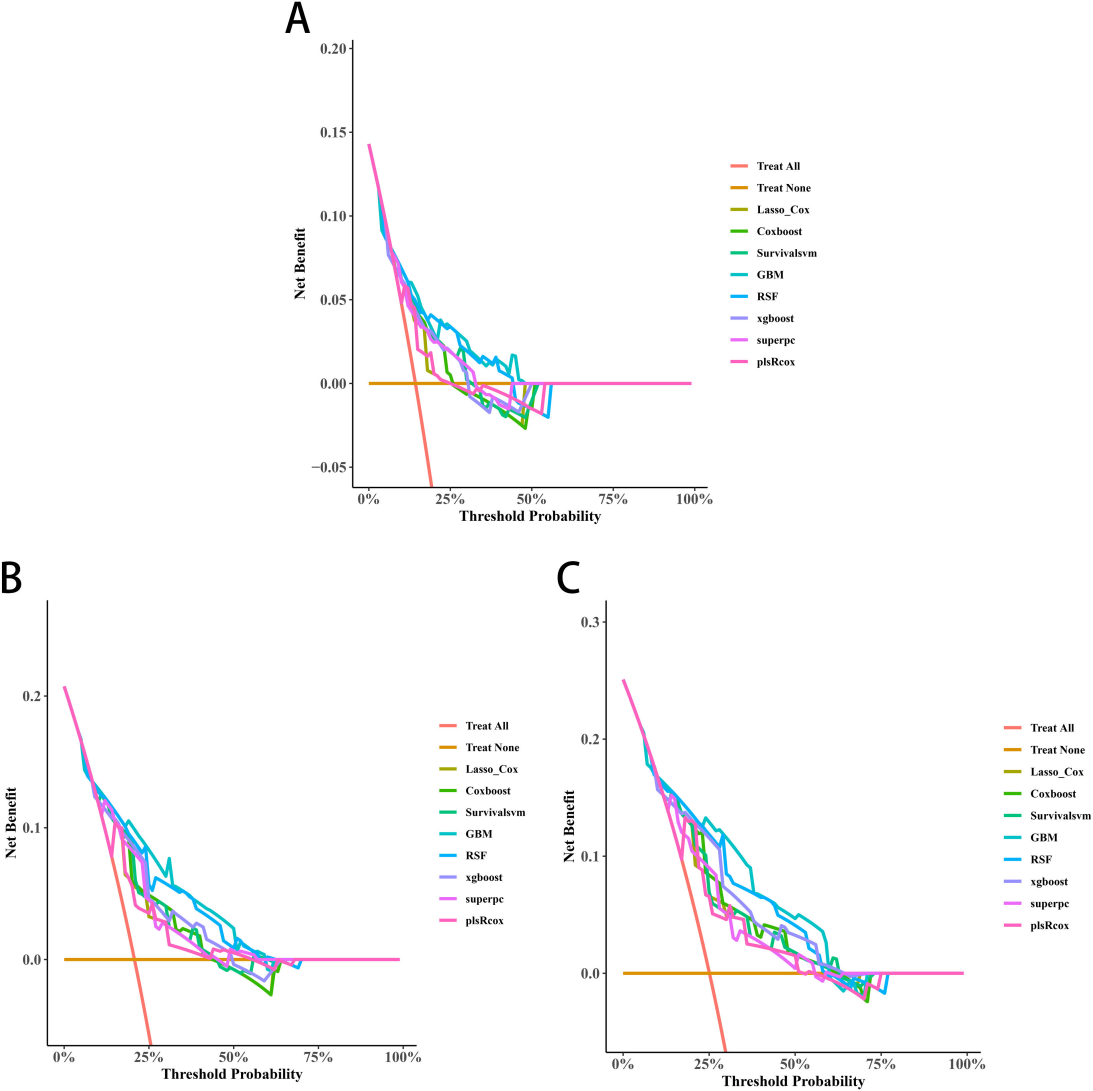
Decision curve analyses (DCAs) of the 3-year **(A)**, 4-year **(B)**, and 5-year BRFS **(C)** prediction models for transition zone prostate cancer.

Three significant covariates (Gleason score, pathological T stage and TET2 mutation status) identified through univariate Cox analysis were included to establish the predictive models. Among the evaluated algorithms, GBM demonstrated optimal predictive performance for 3-year (AUC=0.752, 95%CI:0.619-0.884; **Figure 8A**), 4-year (AUC=0.786, 95%CI:0.675-0.897; **Figure 8B**), and 5-year BRFS (AUC=0.796, 95%CI:0.693-0.898; **Figure 8C**). Clinical applicability was substantiated through DCA (**Figures 9A-C**).

## 4 Discussion

Compared with PCa arising elsewhere in the prostate, PCa occurring in the TZ demonstrates lower Gleason scores and is associated with less aggressive clinical behaviors, thereby leading to a more favorable prognosis (22). The anatomical position of cancer within the prostate may influence treatment decisions and prognostic assessments. However, malignant tissue in the TZ tends to exhibit well-organized neoplastic glandular structures, thereby displaying a differentiated pattern that resembles the morphological characteristics observed in BPH (23–25). This scenario often results in false-negatives in biopsy and imaging outcomes, thus potentially leading to a poorer prognosis (10, 26). In terms of molecular characteristics, some research has explored normal prostate tissue in the TZ; however, genetic profiling studies on PCa occurring in the TZ are limited (23).

In our study, a prominent T>C/A>G mutation accompanied by a less pronounced C>T/G>A mutation was observed only in TZ_PCa6 patient, which may be attributed to tumour heterogeneity. This mutation pattern was defined as signature A, and it was mapped with known signatures in the COSMIC database and exhibited similarity to signature 21. Signature 21, which is associated with microsatellite instability (MSI) and defective DNA mismatch repair, has been observed in stomach and biliary adenocarcinomas (27). Notably, the ubiquitous C>T/G>A mutational pattern (signatures B and C) resembling COSMIC signature 1 likely reflects the interplay of age-related mutagenesis. The predominant mechanism of this mutagenesis involves the spontaneous deamination of 5-methylcytosine at CpG dinucleotides and the mutational burden of signature 1 strongly correlates with age at diagnosis (28, 29). Previous studies have demonstrated that advanced age is a well-established risk factor for the development of prostate cancer (30). As age increases, the gradual decline in cellular DNA repair capacity likely impairs the timely correction of spontaneous deamination of 5-methylcytosine, leading to the accumulation of C>T substitutions. In precancerous lesions, cells under replicative stress exhibit a nearly tenfold increase in C>T substitutions at CpG dinucleotides compared to normal tissues, potentially contributing to prostate cancer initiation (31). Some studies have highlighted age-stratified disparities in prostate cancer prognosis (30, 32, 33). Signature 1 mutations are more common enriched in younger-onset cancers compared to older-onset cohorts, positioning this clock-like signature as a potential biomarker for temporally reconstructing onset of prostate cancer (34). By correlating its mutational burden with cumulative lifelong DNA damage, this signature offers a mechanistic framework to guide subtype-specific diagnostic strategies in prostate cancer.

PCa cells in the TZ acquire a variety of alterations during the course of gene mutations. Driver genes play indispensable roles in cancer occurrence and evolution, whereby they provide cancer cells with the advantage of selective growth. In our study, eight driver mutations (TET2, CDK12, ARID2, CEP89, NKX2-1, ASPSCR1, SPEG, and HOXC11) were identified in PCa occurring in the TZ. TET2, which is a critical gene involved in androgen receptor (AR) signaling exhibits reduced expression in prostate cancer and is associated with adverse clinical outcomes, including elevated Gleason scores and increased metastatic potential (35, 36). CDK12, a cyclin-dependent kinase that directly regulates transcription and controls genomic stability, has been linked to poor prognosis in prostate cancer patients for whom both CDK12 alleles are inactivated, particularly in response to taxane and hormonal therapies (37, 38). Additionally, PCa patients with CDK12 deficiencies may benefit from immune checkpoint immunotherapy and be more responsive to PD-1 inhibitors (39). ARID2 is a member of the AT-rich interactive domain (ARID) family, which affects diverse cancer types, including gastric cancer, PCa, oral squamous cell carcinoma non-small cell lung cancer, sporadic rectal cancer and hepatocellular carcinoma (40–43). During the advancement of stem cells in PCa, ARID2 functions as a PBAF (SWI/SNF-B) chromatin remodeling complex (40). In neuroendocrine prostate cancer, NKX2-1 acts as a key transcriptional driver that upregulates serine/glycine synthesis enzymes, thereby reprogramming cellular metabolism to support proliferation, invasion, and overall tumor aggressiveness under nutrient‐limited conditions (44). HOXC11, a highly conserved transcription factor, is involved in prostate cancer progression, potentially regulated through the hsa_circ_0075542/miR-1197 axis, where miR-1197 promotes tumorigenesis, and HOXC11 may mediate its effects on malignancy (45). ASPSCR1, through its fusion with TFE3, plays a pivotal role in the development of alveolar soft part sarcoma by acting as an aberrant transcriptional transactivator, leading to deregulated transcription and oncogenesis (46). Striated Muscle Enriched Protein Kinase (SPEG) is a serine/threonine kinase predominantly expressed in striated muscles, playing a crucial role in muscle cell cytoskeletal development and cardiac function regulation (47). Decreased SPEG protein levels increase the susceptibility to development of atrial fibrillation (AF), which might become a target for AF treatment (48). Current research on SPEG, ASPSCR1, and CEP89 in prostate cancer is relatively limited. Some studies have shown that CEP89 is upregulated in ovarian cancer, suggesting its potential as a therapeutic target (49). Additionally, genetic defects in CEP89 are associated with autosomal dominant polycystic kidney disease (ADPKD), providing new insights into its molecular pathogenesis (50). Given these findings, further investigation into the role of CEP89 in prostate cancer may reveal novel oncogenic mechanisms and potential therapeutic strategies.

It should be noted that TET2 was validated as a driver gene and demonstrated to be an independent predictor of reduced OS and BRFS in TZ PCa through our cohort analysis using Kaplan-Meier curves with log-rank testing and Cox proportional hazards regression models. The machine learning-driven GBM models consistently demonstrated superior discriminative accuracy (AUC: 0.752-0.915) compared to conventional methods in predicting both OS and BRFS, primarily attributed to their ability to resolve nonlinear interactions between TET2 mutations and traditional clinicopathological parameters. By integrating TET2 status into interpretable nomograms with validated clinical utility via DCA, these computational frameworks establish TET2 not merely as a biomarker but as a dynamic predictor capable of refining personalized risk stratification in TZ PCa. Previous clinical studies also proved that reduced TET2 expression is correlated with advanced Gleason scores, metastatic disease, and poor prognosis and serves as an independent prognostic marker in PCa (35, 36). As a key enzyme that regulates DNA hydroxymethylation (5-hmC), TET2 is intricately linked to AR signaling, which represses AR transcriptional activity by modulating global 5-hmC levels and that its downregulation by androgen signaling enhances AR sensitivity, thus promoting castration-resistant prostate cancer (CRPC) progression (36, 51). In metastatic castration-resistant prostate cancer (mCRPC) therapy development, BET inhibitors are prioritized for their hypothesized role in suppressing c-MYC and enhancing AR pathway engagement (52). Kong et al. demonstrated that bromodomain and extra-terminal (BET) inhibitors downregulate TET2 expression, thereby enhancing the antitumor activity of chimeric antigen receptor (CAR) T cells (53). These findings unveil that BET inhibition may counteract TET2-mediated epigenetic reprogramming through immune contexture remodeling in prostate cancer. DOT1L, which is a histone H3K79 methyltransferase, promotes oncogenic transcription in leukemia and solid tumors (including PCa) by maintaining H3K79me2 at proliferative genes (54, 55). Although the direct associations between TET2 and DOT1L have not been fully elucidated, both enzymes regulate overlapping epigenetic landscapes. The loss of TET2 results in elevated H3K79 dimethylation, thereby leading to these cells being more reliant on DOT1L activity (55, 56). Furthermore, the noncanonical role of TET2 in suppressing mTORC1 via urea cycle modulation may intersect with the metabolic functions of DOT1L, thereby increasing the degree of vulnerability exploitable through dual inhibition (57, 58).

Furthermore, our study revealed AR mutations at X:66765174 (in-frame insertion) and X:66931247 (missense mutation, W741C) in TZ PCa. The observed mutations may contribute to resistance against leuprolide (which is a luteinizing hormone-releasing hormone analogue) and bicalutamide (which is a first-generation antiandrogen). Emerging evidence underscores the critical role of androgen receptor (AR) mutations in driving therapeutic resistance in prostate cancer. Hara et al. (2005) proposed that hypermutational states may promote clonal selection of AR variants, which is exemplified by the W741C mutation (position 2223); this mechanism confers bicalutamide resistance by converting this antagonist into an AR agonist via structural alterations in the ligand-binding domain (59). This mechanism aligns with clinical observations by Grossmann et al., who identified treatment-emergent AR mutations as key drivers of acquired resistance to androgen-deprivation therapies in advanced disease (60). Hingorani et al. further demonstrated that W741C-harbouring xenografts retain sensitivity to flutamide (despite bicalutamide resistance), thus demonstrating the drug-specific nature of AR mutations (61). These findings were corroborated by Ledet et al., who conducted a large-scale ctDNA analysis and confirmed recurrent AR mutations (such as W741C and T878A) as biomarkers predictive of bicalutamide resistance (62). Collectively, these studies emphasize the necessity of profiling AR mutations to guide therapeutic decisions and monitor adaptive responses in both treatment-naïve and castration-resistant prostate cancer patients (63). However, regarding leuprolide resistance, current evidence remains limited. To date, no large-scale cohort studies have explicitly correlated AR mutations with leuprolide resistance in prostate cancer. Future research should focus on conducting larger-scale cohort studies and in-depth molecular analyses to explore the relationship between AR mutations and leuprolide resistance, which could provide a basis for developing personalized treatment strategies and improving patient outcomes.

Several study limitations should be considered. First, the restricted sample availability in whole-exome sequencing compromised our ability to fully mitigate false discovery rates stemming from multiple hypothesis testing, thereby necessitating additional investigation through orthogonal biological assays to substantiate the proposed molecular mechanisms. Second, while Kaplan-Meier analyses, Cox regression methodologies, and machine learning algorithms were rigorously applied, the relatively limited patient cohort may lead to potential selection bias, which necessitates future validation in larger, multicentric populations to explore the connection between TET2 mutant status and prognosis of TZ PCa patients. Lastly, in this study, no molecular biology experiments involving primary cells were conducted to explore the role and molecular mechanisms of driver genes in the malignant progression of TZ PCa. The rarity of TZ PCa inherently restricts the acquisition of viable primary cell cultures from freshly resected specimens, necessitating alternative methodological approaches for robust mechanistic studies. In addition, established prostate cancer cell lines such as PC3, DU145, 22RV1, and LNCaP exhibit inherent limitations in recapitulating the pathophysiological profiles of TZ PCa, particularly regarding tumor-stroma interactions and metastatic evolution patterns.

## 5 Conclusions

By employing state-of-the-art whole-exome sequencing to analyze the genetic profiles of TZ PCa samples, we have enhanced the understanding of the disease. This knowledge facilitates timely identification of patients through improved diagnostic effectiveness, ultimately optimizing clinical prognosis. TET2 gene mutation status may serve as an independent predictor of reduced OS and BRFS in TZ PCa patients. Integration of TET2 status into machine learning-based prognostic models significantly enhances predictive accuracy and provides refined risk stratification for personalized management. Future multi-center studies in prospective cohorts are required to validate the external validity of these findings across diverse clinical settings.

## Data Availability

The data presented in the study are deposited in the FigShare repository, DOI: TZ_PCa1: 10.6084/m9.figshare.28689293 TZ_PCa2: 10.6084/m9.figshare.28689731 TZ_PCa3: 10.6084/m9.figshare.28689737 TZ_PCa4: 10.6084/m9.figshare.28689749 TZ_PCa5: 10.6084/m9.figshare.28689755 TZ_PCa6: 10.6084/m9.figshare.28689761 TZ_PCa7: 10.6084/m9.figshare.28689764 TZ_PCa8: 10.6084/m9.figshare.28689776 TZ_PCa9: 10.6084/m9.figshare.28689779.
