# Correction: TET2 gene mutation status associated with poor prognosis of transition zone prostate cancer: a retrospective cohort study based on whole exome sequencing and machine learning models

**DOI:** 10.3389/fendo.2025.1738698

**Published:** 2025-12-10

**Authors:** Yutong Wang, Ailing Yu, Ziping Gao, Xiaoying Yuan, Xiaochen Du, Peng Shi, Haoyun Guan, Shuang Wen, Honglong Wang, Liang Wang, Bo Fan, Zhiyu Liu

**Affiliations:** 1 Department of Urology, Second Affiliated Hospital of Dalian Medical University, Dalian, Liaoning, China; 2 Liaoning Provincial Key Laboratory of Urological Digital Precision Diagnosis and Treatment, Second Affiliated Hospital of Dalian Medical University, Dalian, Liaoning, China; 3 Department of Urology, Liaoning Engineering Research Center of Integrated Precision Diagnosis and Treatment Technology for Urological Cancer, Dalian, Liaoning, China; 4 Dalian Key Laboratory of Prostate Cancer Research, Second Affiliated Hospital of Dalian Medical University, Dalian, Liaoning, China; 5 Department of Colorectal Surgery, Dalian Municipal Central Hospital, Dalian, Liaoning, China; 6 First Clinical College, Dalian Medical University, Dalian, Liaoning, China; 7 Department of Anatomy, College of Basic Medicine, Dalian Medical University, Dalian, Liaoning, China; 8 College of Humanities and Social Sciences, Dalian Medical University, Dalian, Liaoning, China; 9 Second Clinical College, Dalian Medical University, Dalian, Liaoning, China; 10 Department of Pathology, Dalian Friendship Hospital, Dalian, China

**Keywords:** transition zone, prostate cancer, whole-exome sequencing, driver genes, medication prediction, TET2 mutation, machine learning models

There was a mistake in **Figure 4A** as published. The image in **Figure 4A** was inadvertently duplicated in **Figure 7A** due to a technical oversight during the figure assembly process. The corrected **Figure 4A** appears below.

**Figure 4:**
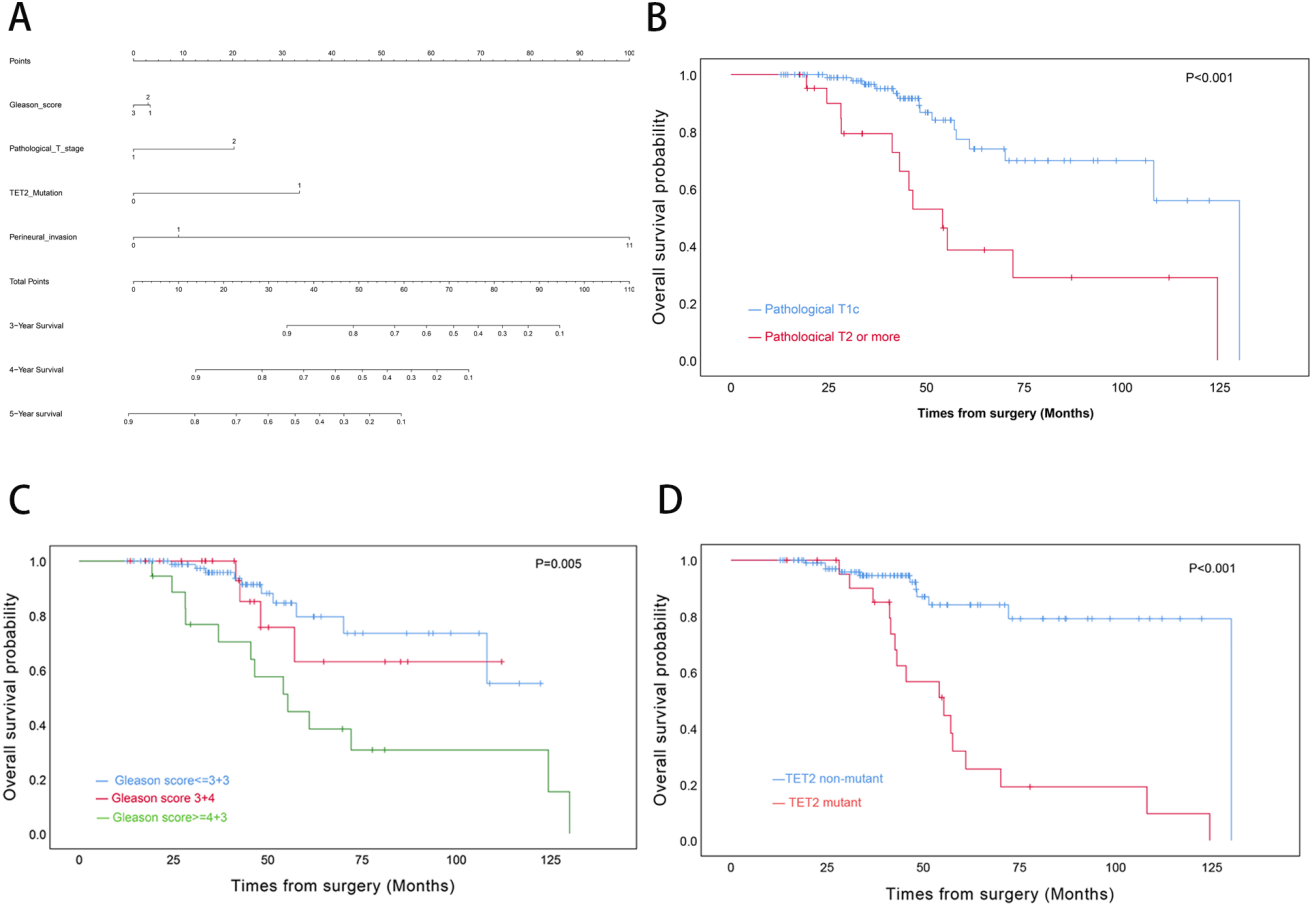
Prognostic modeling and survival analysis in transition zone prostate cancer. **(A)** Nomogram integrating clinicopathological variables for predicting 3-, 4-, and 5-year overall survival probabilities. **(B)** Kaplan-Meier curves stratified by pathological T stage. **(C)** Survival differentiation across Gleason grade groups. **(D)** Comparative survival analysis by TET2 mutation status.

The original version of this article has been updated.

