# Supplementary material for: TET2 gene mutation status associated with poor prognosis of transition zone prostate cancer: a retrospective cohort study based on whole exome sequencing and machine learning models
Source: Front Endocrinol (Lausanne). 2025 Apr 14;16:1568665. doi: 10.3389/fendo.2025.1568665 (PMC12034557; doi:10.3389/fendo.2025.1568665)
Supplement: Supplementary file 1 [file DataSheet1.docx]

Supplementary Material

# **Supplementary Figures and Tables**

## **1.1 Supplementary Figures**

**Supplementary Figure S1** Distribution of single nucleotide polymorphisms in nine samples. The images display the number of single nucleotide polymorphisms in various genomic regions (left) and coding regions (right) from TZ_PCa1 to TZ_PCa9 (A-I). CDS: coding sequence, UTR: untranslated region.


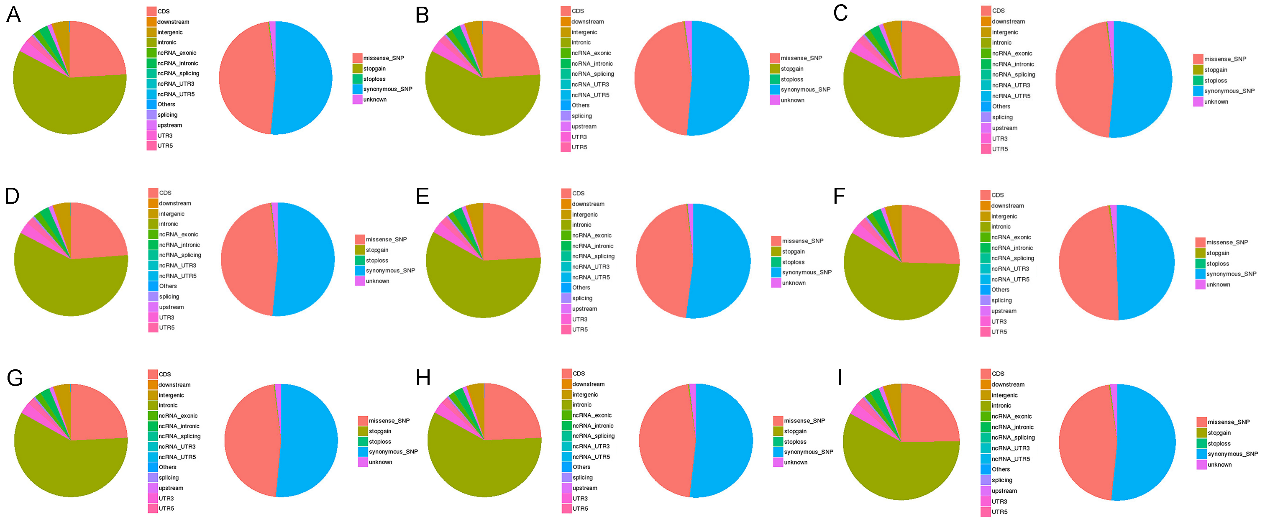


**Supplementary Figure S2** Distribution of insertions and deletions in nine samples. The images display the number of insertions and deletions in various genomic regions (left) and coding regions (right) from TZ_PCa1 to TZ_PCa9 (A-I). CDS: coding sequence, UTR: untranslated region.


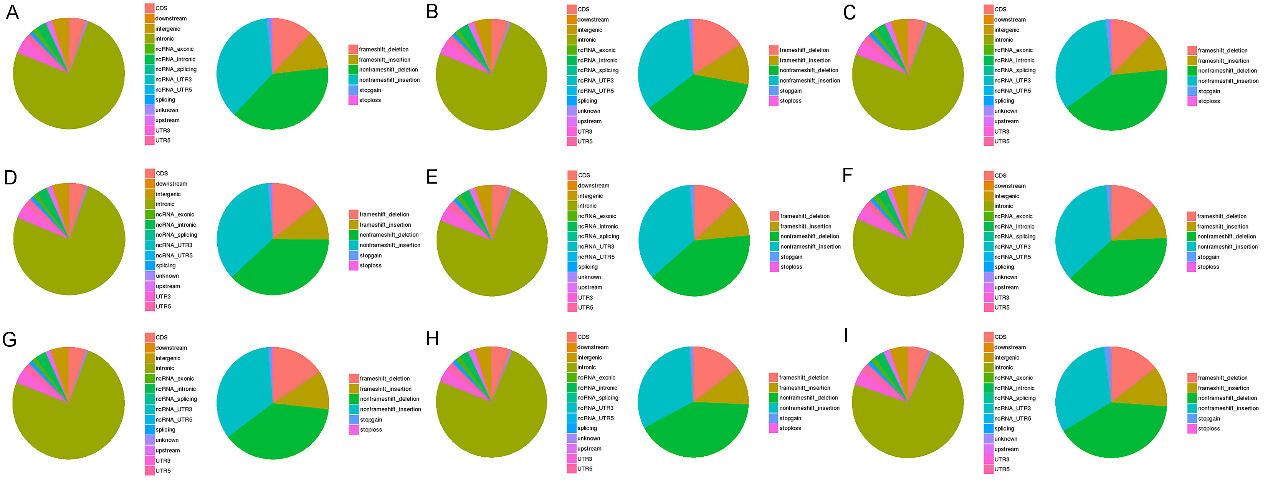


**Supplementary Figure S3** Mutation spectrum analysis illustrates the proportions of different types of variants in nine TZ PCa.


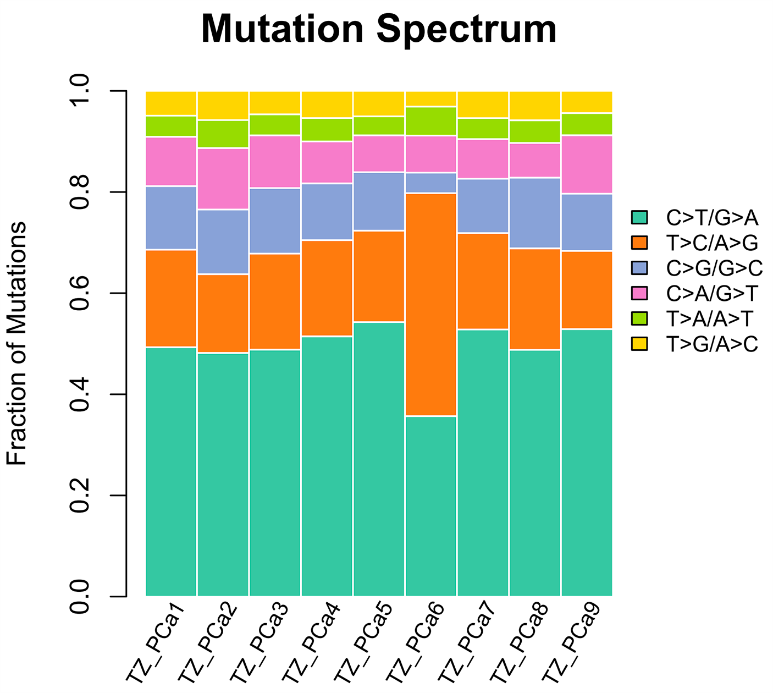


**Supplementary Figure S4** Cosine similarities of mutation signatures in TZ PCa and 30 known mutation signatures. Clustering analysis was performed on mutation signatures in nine samples and 30 known mutation signatures (A). The cosine similarities of mutation signatures are presented in the heatmap (B).


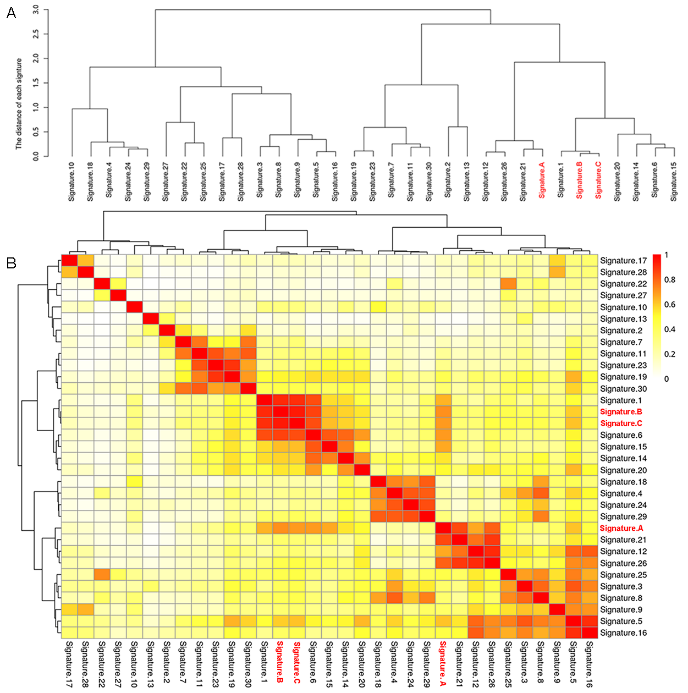


**Supplementary Figure S5** The mutation landscape of the eight driver genes (TET2, CDK12, CEP89, NKX2-1, ASPSCR1, ARID2, SPEG, and HOXC11) in TZ PC.


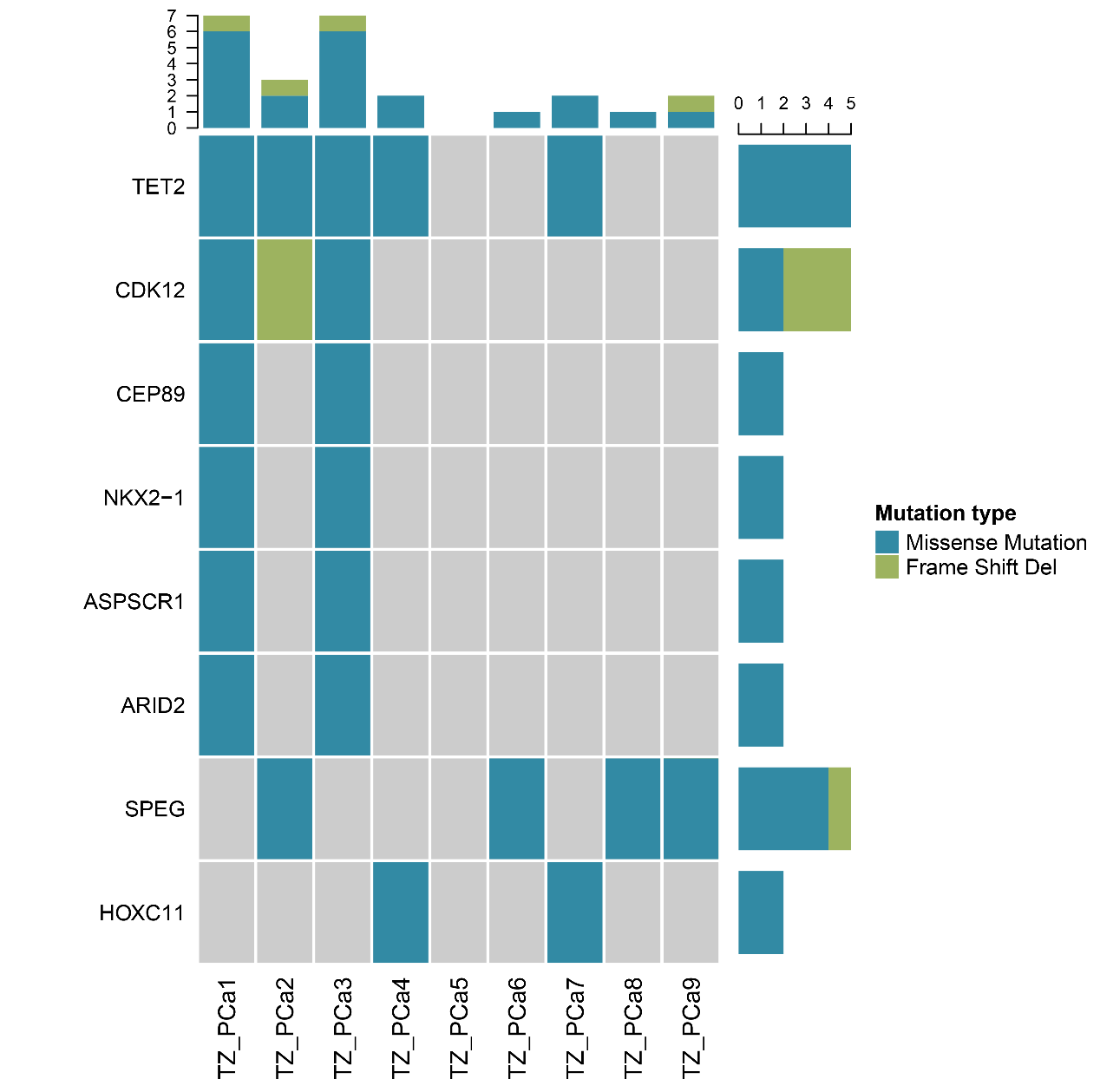


**Supplementary Figure S6** Significantly mutated genes, including somatic SNVs, INDELs and other mutations, are displayed in the heatmap.


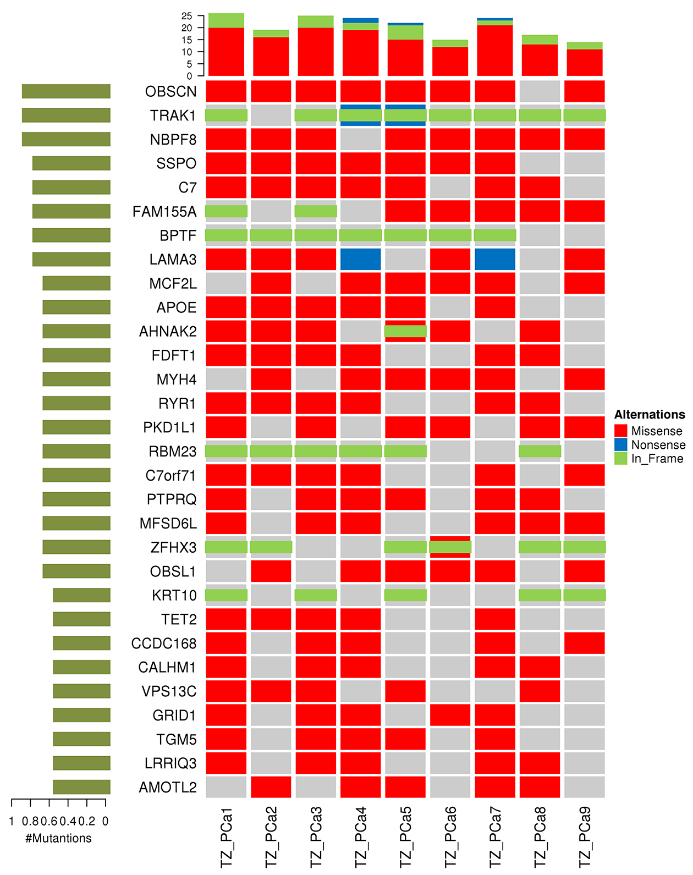


## **Supplementary Tables**

**Supplementary Table 1.** Nine cases of prostate cancer in transition zone.

| Patient | Age | PSA at diagnosis (ng/ml) | fPSA at diagnosis (ng/ml) | PSAD at diagnosis (ng/ml^2^) | Gleason score | TNM stage | Treatment | Follow-up (month) | Outcome |
| --- | --- | --- | --- | --- | --- | --- | --- | --- | --- |
| TZ_PCa1 | 82 | 23.11 | 3 | 0.61 | 3+3=6 | T2aN0M0 | LRP | 125.8 | Alive |
| TZ_PCa2 | 64 | 7.19 | 2.38 | 0.16 | 3+4=7 | T2bN0M0 | LRP | 17.2 | Alive |
| TZ_PCa3 | 86 | 20.01 | 2.53 | NA | 3+4=7 | T2aN0M0 | LRP | 55.9 | Alive |
| TZ_PCa4 | 85 | 3.33 | 0.589 | 0.43 | 5+4=9 | T2aN0M0 | LRP | 23.1 | Death |
| TZ_PCa5 | 75 | 9.54 | 1.71 | 0.29 | 3+3=6 | T2aN0M0 | TURP+ADT | 12.4 | Alive |
| TZ_PCa6 | 69 | 18.45 | 2.76 | 0.34 | 3+3=6 | T2aN0M0 | LRP | 13.8 | Alive |
| TZ_PCa7 | 75 | 17.1 | 3.71 | 0.11 | 3+3=6 | T1bN0M0 | TURP+ADT | 25.5 | Alive |
| TZ_PCa8 | 76 | 3.45 | 0.82 | 0.12 | 3+4＝7 | T2aN0M0 | LRP | 54.5 | Alive |
| TZ_PCa9 | 81 | 2.44 | 0.58 | 0.07 | 4+4＝8 | T2aN0M0 | LRP | 58.1 | Alive |

1. NA: Not available, PSA: Prostate-specific antigen; LRP: Laparoscopic radical prostatectomy; PSAD: Prostate specific antigen density; TURP: Transurethral resection of prostate; ADT: Androgen deprivation therapy.

**Supplementary Table** **2**. The count of SNPs in various genomic and coding regions.

| **Sample** | **TZ_PCa1** | **TZ_PCa2** | **TZ_PCa3** | **TZ_PCa4** | **TZ_PCa5** | **TZ_PCa6** | **TZ_PCa7** | **TZ_PCa8** | **TZ_PCa9** |
| --- | --- | --- | --- | --- | --- | --- | --- | --- | --- |
| CDS | 21547 | 21133 | 21597 | 21675 | 21429 | 23847 | 21560 | 21337 | 21122 |
| Synonymous SNP | 11073 | 10861 | 11091 | 11169 | 11154 | 11802 | 11101 | 11048 | 10912 |
| Missense SNP | 10002 | 9773 | 10036 | 10036 | 9856 | 11485 | 9995 | 9796 | 9709 |
| Stopgain | 69 | 71 | 69 | 67 | 61 | 111 | 69 | 72 | 75 |
| Stoploss | 12 | 6 | 12 | 7 | 8 | 9 | 6 | 10 | 6 |
| Unknown | 391 | 422 | 389 | 396 | 350 | 440 | 389 | 411 | 420 |
| Intronic | 52624 | 51860 | 52528 | 53556 | 52317 | 54669 | 52401 | 51474 | 49488 |
| UTR3 | 3066 | 2994 | 3064 | 3050 | 2978 | 3065 | 2960 | 2952 | 2838 |
| UTR5 | 1933 | 1865 | 1955 | 1974 | 1929 | 1950 | 1841 | 1821 | 1839 |
| Splicing | 491 | 515 | 497 | 509 | 495 | 623 | 505 | 487 | 490 |
| ncRNA exonic | 1638 | 1671 | 1637 | 1736 | 1501 | 1671 | 1670 | 1616 | 1552 |
| ncRNA intronic | 2416 | 2333 | 2397 | 2519 | 2399 | 2437 | 2439 | 2340 | 2225 |
| ncRNA splicing | 14 | 15 | 17 | 19 | 10 | 16 | 19 | 17 | 14 |
| Upstream | 1144 | 1145 | 1142 | 1154 | 1077 | 1116 | 1043 | 1069 | 1072 |
| Downstream | 491 | 504 | 484 | 485 | 448 | 474 | 457 | 469 | 463 |
| Intergenic | 3945 | 3786 | 3946 | 3990 | 4219 | 3914 | 3995 | 4099 | 4103 |
| Others | 98 | 102 | 98 | 105 | 88 | 79 | 92 | 105 | 89 |
| Total | 89565 | 88132 | 89515 | 91001 | 88585 | 93893 | 88977 | 87632 | 84978 |

SNPs: single nucleotide polymorphisms, CDS: coding sequence, UTR: untranslated region, ncRNA: noncoding ribonucleic acid.

**Supplementary Table 3.** The count of SNPs in different regions of genome.

| **Sample** | **TZ_PCa1** | **TZ_PCa2** | **TZ_PCa3** | **TZ_PCa4** | **TZ_PCa5** | **TZ_PCa6** | **TZ_PCa7** | **TZ_PCa8** | **TZ_PCa9** |
| --- | --- | --- | --- | --- | --- | --- | --- | --- | --- |
| Total | 89565 | 88132 | 89515 | 91001 | 88585 | 93893 | 88977 | 87632 | 84978 |
| Het | 51679 | 50102 | 51624 | 51803 | 51021 | 56823 | 50605 | 50489 | 50772 |
| Hom | 37886 | 38030 | 37891 | 39198 | 37564 | 37070 | 38372 | 37143 | 34206 |
| Transition | 62958 | 62037 | 62922 | 63983 | 62439 | 66995 | 62616 | 61677 | 60008 |
| Transvertion | 26607 | 26095 | 26593 | 27018 | 26146 | 26898 | 26361 | 25955 | 24970 |
| TS/TV | 2.37 | 2.38 | 2.37 | 2.37 | 2.39 | 2.49 | 2.38 | 2.38 | 2.40 |
| dbSNP percentage | 88908 (99.27%) | 87458 (99.24%) | 88854 (99.26%) | 90451  (99.40%) | 87867  (99.19%) | 88794 (94.57%) | 88432  (99.39%) | 86976 (99.25%) | 83461 (98.21%) |
| Novel | 657 | 674 | 661 | 550 | 718 | 5099 | 545 | 656 | 1517 |
| Novel TS | 398 | 403 | 406 | 357 | 462 | 4156 | 364 | 386 | 1061 |
| Novel TV | 259 | 271 | 255 | 193 | 256 | 943 | 181 | 270 | 456 |
| Novel TS/TV | 1.54 | 1.49 | 1.59 | 1.85 | 1.80 | 4.41 | 2.01 | 1.43 | 2.33 |

SNPs: single nucleotide polymorphism, Het: heterozygote, Hom: homozygote, TS: transition, TV: transversion, dbSNP: the single nucleotide polymorphism.

**Supplementary Table 4.** The count of INDELs in various genomic and coding regions.

| **Sample** | **TZ_PCa1** | **TZ_PCa2** | **TZ_PCa3** | **TZ_PCa4** | **TZ_PCa5** | **TZ_PCa6** | **TZ_PCa7** | **TZ_PCa8** | **TZ_PCa9** |
| --- | --- | --- | --- | --- | --- | --- | --- | --- | --- |
| CDS | 604 | 592 | 591 | 651 | 615 | 635 | 584 | 636 | 663 |
| Frameshift deletion | 63 | 81 | 63 | 81 | 70 | 78 | 79 | 81 | 84 |
| Frameshift insertion | 59 | 61 | 57 | 61 | 56 | 55 | 56 | 61 | 68 |
| Nonframeshift deletion | 204 | 188 | 212 | 214 | 215 | 214 | 192 | 230 | 233 |
| Nonframeshift insertion | 191 | 172 | 171 | 200 | 188 | 194 | 171 | 175 | 183 |
| Stopgain | 7 | 6 | 7 | 6 | 7 | 8 | 5 | 5 | 11 |
| Stoploss | 2 | 2 | 2 | 2 | 0 | 0 | 2 | 1 | 1 |
| Unknown | 78 | 82 | 79 | 87 | 79 | 86 | 79 | 83 | 83 |
| Intronic | 9068 | 8989 | 8655 | 10063 | 8833 | 9075 | 8741 | 8771 | 7952 |
| UTR3 | 497 | 482 | 484 | 556 | 500 | 507 | 491 | 488 | 464 |
| UTR5 | 276 | 268 | 281 | 314 | 309 | 277 | 267 | 281 | 300 |
| Splicing | 134 | 136 | 139 | 159 | 155 | 142 | 147 | 143 | 133 |
| ncRNA exonic | 160 | 148 | 153 | 164 | 157 | 174 | 150 | 168 | 154 |
| ncRNA intronic | 371 | 372 | 348 | 422 | 329 | 343 | 364 | 325 | 317 |
| ncRNA splicing | 4 | 4 | 4 | 4 | 2 | 2 | 4 | 4 | 3 |
| Upstream | 215 | 224 | 209 | 222 | 208 | 199 | 176 | 225 | 202 |
| Downstream | 76 | 85 | 74 | 84 | 85 | 74 | 70 | 74 | 70 |
| Intergenic | 536 | 535 | 504 | 583 | 519 | 525 | 541 | 516 | 513 |
| Others | 13 | 14 | 16 | 22 | 15 | 10 | 15 | 14 | 14 |
| Total | 11954 | 11849 | 11458 | 13244 | 11727 | 11963 | 11550 | 11645 | 10785 |

CDS: Coding sequence, UTR: untranslated region, ncRNA: noncoding ribonucleic acid.

**Supplementary Table 5.** The count of INDELs in different regions of the genome.

| **Sample** | **TZ_PCa1** | **TZ_PCa2** | **TZ_PCa3** | **TZ_PCa4** | **TZ_PCa5** | **TZ_PCa6** | **TZ_PCa7** | **TZ_PCa8** | **TZ_PCa9** |
| --- | --- | --- | --- | --- | --- | --- | --- | --- | --- |
| Total | 11954 | 11849 | 11458 | 13244 | 11727 | 11963 | 11550 | 11645 | 10785 |
| Het | 6247 | 6141 | 5868 | 6960 | 6138 | 6288 | 5907 | 6093 | 5935 |
| Hom | 5707 | 5708 | 5590 | 6284 | 5589 | 5675 | 5643 | 5552 | 4850 |
| dbSNP percentage | 10431 (87.26%) | 10250 (86.51%) | 10212 (89.13%) | 11167 (84.32%) | 10107 (86.19%) | 10114 (84.54%) | 10210 (88.40%) | 10107 (86.79%) | 9180 (85.12%) |
| Novel | 1523 | 1599 | 1246 | 2077 | 1620 | 1849 | 1340 | 1538 | 1605 |

INDELs: insertions and deletions, Het: heterozygote, Hom: homozygote, dbSNP: the single nucleotide polymorphism.

**Supplementary Table 6**. The number of somatic SNVs in different regions of the genome.

| **Sample** | **TZ_PCa1** | **TZ_PCa2** | **TZ_PCa3** | **TZ_PCa4** | **TZ_PCa5** | **TZ_PCa6** | **TZ_PCa7** | **TZ_PCa8** | **TZ_PCa9** |
| --- | --- | --- | --- | --- | --- | --- | --- | --- | --- |
| CDS | 430 | 469 | 432 | 410 | 398 | 1676 | 409 | 379 | 433 |
| Missense SNP | 423 | 463 | 425 | 402 | 397 | 1636 | 401 | 373 | 422 |
| Stopgain | 7 | 6 | 7 | 8 | 1 | 36 | 8 | 5 | 11 |
| Stoploss | 0 | 0 | 0 | 0 | 0 | 4 | 0 | 1 | 0 |
| Total | 430 | 469 | 432 | 410 | 398 | 1676 | 409 | 379 | 433 |

SNVs: single nucleotide variants, CDS: Coding sequence, SNP: single nucleotide polymorphism.

**Supplementary Table 7.** Somatic INDELs in different regions of the genome.

| **Sample** | **TZ_PCa1** | **TZ_PCa2** | **TZ_PCa3** | **TZ_PCa4** | **TZ_PCa5** | **TZ_PCa6** | **TZ_PCa7** | **TZ_PCa8** | **TZ_PCa9** |
| --- | --- | --- | --- | --- | --- | --- | --- | --- | --- |
| CDS | 59 | 48 | 48 | 74 | 68 | 76 | 52 | 65 | 97 |
| Frameshift deletion | 4 | 13 | 3 | 11 | 7 | 13 | 10 | 6 | 18 |
| Frameshift insertion | 3 | 3 | 3 | 4 | 2 | 6 | 3 | 7 | 6 |
| Nonframeshift deletion | 16 | 16 | 28 | 23 | 21 | 21 | 19 | 25 | 37 |
| Nonframeshift insertion | 36 | 14 | 14 | 34 | 34 | 35 | 19 | 27 | 31 |
| Stopgain | 0 | 1 | 0 | 2 | 4 | 1 | 1 | 0 | 5 |
| Stoploss | 0 | 1 | 0 | 0 | 0 | 0 | 0 | 0 | 0 |
| Total | 59 | 48 | 48 | 74 | 68 | 76 | 52 | 65 | 97 |

INDELs: insertions and deletions, CDS: Coding sequence

**Supplementary Table 8.** The results of mutation signature and screening annotation

| Our signature | Near reference signature | Cosine similarity | Correlation coefficient | Cancer types | Proposed etiology | Additional mutational features |
| --- | --- | --- | --- | --- | --- | --- |
| Signature A | Signature 21 | 0.85 | 0.82 | Signature 21 has been found only in stomach cancer | The etiology of Signature 21 remains unknown | N/A |
| Signature B | Signature 1 | 0.91 | 0.90 | Signature 1 has been found in all cancer types and in most cancer samples | Signature 1 is the result of an endogenous mutational process initiated by spontaneous deamination of 5-methylcytosine | Signature 1 is associated with small numbers of small insertions and deletions in most tissue types |
| Signature C | Signature 1 | 0.90 | 0.89 |  |  |  |

**Supplementary Table 9.** Prediction of targeted drug for significantly mutated genes in prostate cancer of transition zone.

| Gene symbol | Entriz ID | Chr | Position | Variant classification | AAChange | Drug name | Drug type | Source |
| --- | --- | --- | --- | --- | --- | --- | --- | --- |
| RYR1 | 6261 | 19 | 38976612 | Missense Mutation | NM_000540:exon34:c.C5317T:p.P1773S | Caffeine | Small molecule | DrugBank |
| RYR1 | 6261 | 19 | 39025366 | Missense Mutation | NM_001042723:exon78:c.C11251G:p.Q3751E | Caffeine | Small molecule | DrugBank |
| RYR1 | 6261 | 19 | 38951178 | Missense Mutation | NM_000540:exon20:c.A2524C:p.S842R | Caffeine | Small molecule | DrugBank |
| APOE | 348 | 19 | 45412079 | Missense Mutation | NM_000041:exon4:c.C526T:p.R176C | Human serum albumin | Biotech | DrugBank |
| TET2 | 54790 | 4 | 106157703 | Missense Mutation | NM_001127208:exon3:c.T2604G:p.F868L | Bromodomain inhibitors | NA | MCG |
| TET2 | 54790 | 4 | 106157703 | Missense Mutation | NM_001127208:exon3:c.T2604G:p.F868L | DOT1L inhibitors | NA | MCG |
| ERCC5 | 2073 | 13 | 103515085 | Missense Mutation | NM_000123:exon8:c.G1586C:p.C529S | Platinum | Efficacy | PharmGKB |
| UQCRC1 | 7384 | 3 | 48636614 | Nonsense Mutation | NM_003365:exon13:c.C1390T:p.Q464X | Myxothiazol | Small molecule | DrugBank |
| UQCRC1 | 7384 | 3 | 48636615 | Missense Mutation | NM_003365:exon13:c.G1389T:p.E463D | Myxothiazol | Small molecule | DrugBank |
| UQCRC1 | 7384 | 3 | 48638451 | Missense Mutation | NM_003365:exon8:c.A923G:p.N308S | Myxothiazol | Small molecule | DrugBank |
| HDC | 3067 | 15 | 50555544 | Missense Mutation | NM_002112:exon2:c.C92T:p.T31M | Pyridoxal phosphate | Small molecule | DrugBank |
| HDC | 3067 | 15 | 50534514 | Missense Mutation | NM_002112:exon12:c.A1932C:p.E644D | Pyridoxal phosphate | Small molecule | DrugBank |
| SERPINE1 | 5054 | 7 | 100771717 | Missense Mutation | NM_000602:exon2:c.G43A:p.A15T | Alteplase | Biotech | DrugBank |
| APOB | 338 | 2 | 21228348 | Missense Mutation | NM_000384:exon26:c.A11392G:p.T3798A | Irbesartan | Efficacy | PharmGKB |
| APOB | 338 | 2 | 21236085 | Missense Mutation | NM_000384:exon25:c.G4163A:p.R1388H | Irbesartan | Efficacy | PharmGKB |
| APOB | 338 | 2 | 21225485 | Missense Mutation | NM_000384:exon29:c.G12809C:p.R4270T | Irbesartan | Efficacy | PharmGKB |
| APOB | 338 | 2 | 21231387 | Missense Mutation | NM_000384:exon26:c.A8353C:p.N2785H | Irbesartan | Efficacy | PharmGKB |
| TRPM8 | 79054 | 2 | 234854540 | Missense Mutation | NM_024080:exon7:c.G740C:p.R247T | Menthol | Small molecule | DrugBank |
| TRPM8 | 79054 | 2 | 234854552 | Missense Mutation | NM_024080:exon7:c.A752G:p.Y251C | Menthol | Small molecule | DrugBank |
| CACNA1A | 773 | 19 | 13409890 | Missense Mutation | NM_001127221:exon19:c.C2560T:p.R854C | Pregabalin | Small molecule | DrugBank |
| CACNA1A | 773 | 19 | 13318703 | In Frame Insertion | NM_001127222:exon47:c.6944_6945insACA:  p.Q2315delinsQQ | Pregabalin | Small molecule | DrugBank |
| CACNA1A | 773 | 19 | 13318674 | In Frame Insertion | NM_001127222:exon47:c.6973_6974insTGC:  p.Q2325delinsLQ | Pregabalin | Small molecule | DrugBank |
| CACNA1A | 773 | 19 | 13318672 | In Frame Deletion | NM_001127222:exon47:c.6964_6975del:  p.2322_2325del | Pregabalin | Small molecule | DrugBank |
| IGF2R | 3482 | 6 | 160491079 | Missense Mutation | NM_000876:exon31:c.G4432A:p.E1478K | Mecasermin | Biotech | DrugBank |
| IGF2R | 3482 | 6 | 160524825 | Missense Mutation | NM_000876:exon47:c.A7043G:p.N2348S | Mecasermin | Biotech | DrugBank |
| GSTM5 | 2949 | 1 | 110257814 | Missense Mutation | NM_000851:exon7:c.G519C:p.K173N | Glutathione | Small molecule | DrugBank |
| MUT | 4594 | 6 | 49415448 | Missense Mutation | NM_000255:exon8:c.G1495A:p.A499T | Cyanocobalamin | Small molecule | DrugBank |
| NISCH | 11188 | 3 | 52522246 | Missense Mutation | NM_007184:exon16:c.C2738T:p.T913M | Tizanidine | Small molecule | DrugBank |
| NISCH | 11188 | 3 | 52526184 | Frame Shift Deletion | NM_007184:exon21:c.4202_4203del:p.Q1401fs | Tizanidine | Small molecule | DrugBank |
| ADCY2 | 108 | 5 | 7826853 | Missense Mutation | NM_020546:exon25:c.G3145A:p.V1049I | Forskolin | Small molecule | DrugBank |
| NCAN | 1463 | 19 | 19329924 | Missense Mutation | NM_004386:exon3:c.C274T:p.P92S | Thiodigalactoside | Small molecule | DrugBank |
| PYGB | 5834 | 20 | 25263834 | Missense Mutation | NM_002862:exon13:c.C1541G:p.T514S | Pyridoxal phosphate | Small molecule | DrugBank |
| RAMP3 | 10268 | 7 | 45217015 | Missense Mutation | NM_005856:exon2:c.T166C:p.W56R | Pramlintide | Biotech | DrugBank |
| KCNN2 | 3781 | 5 | 113698638 | In Frame Insertion | NM_021614:exon1:c.166_167insCCA:p.A56delinsAT | Miconazole | Small molecule | DrugBank |
| KCNN2 | 3781 | 5 | 113698632 | In Frame Insertion | NM_021614:exon1:c.160_161insCCA:p.A54delinsAT | Miconazole | Small molecule | DrugBank |
| KCNN2 | 3781 | 5 | 113698634 | In Frame Insertion | NM_021614:exon1:c.162_163insGCA:p.A54delinsAA | Miconazole | Small molecule | DrugBank |
| KCNN2 | 3781 | 5 | 113698638 | In Frame Insertion | NM_021614:exon1:c.166_167insCCT:p.A56delinsAS | Miconazole | Small molecule | DrugBank |
| KCNN2 | 3781 | 5 | 113698640 | In Frame Insertion | NM_021614:exon1:c.168_169insGCT:p.A56delinsAA | Miconazole | Small molecule | DrugBank |
| BST1 | 683 | 4 | 15709192 | Missense Mutation | NM_004334:exon3:c.G374A:p.R125H | Nicotinamide | Small molecule | DrugBank |
| TAS1R2 | 80834 | 1 | 19166893 | Missense Mutation | NM_152232:exon6:c.G1720A:p.A574T | Aspartame | Small molecule | DrugBank |
| IL4R | 3566 | 16 | 27374139 | Missense Mutation | NM_001257406:exon10:c.T1466C:p.L489P | Pitrakinra | Efficacy | PharmGKB |
| IL4R | 3566 | 16 | 27373964 | Missense Mutation | NM_001257406:exon10:c.T1291C:p.C431R | Pitrakinra | Efficacy | PharmGKB |
| HCAR3 | 8843 | 12 | 123201233 | Missense Mutation | NM_006018:exon1:c.T52C:p.C18R | Niacin | Small molecule | DrugBank |
| HCAR3 | 8843 | 12 | 123200158 | Missense Mutation | NM_006018:exon1:c.C1127G:p.A376G | Niacin | Small molecule | DrugBank |
| IGFBP3 | 3486 | 7 | 45956969 | Missense Mutation | NM_000598:exon2:c.A473C:p.H158P | Mecasermin | Biotech | DrugBank |
| ITGAL | 3683 | 16 | 30528973 | Missense Mutation | NM_001114380:exon25:c.C2728T:p.P910S | Efalizumab | Biotech | DrugBank |
| ITGAL | 3683 | 16 | 30531233 | Missense Mutation | NM_001114380:exon28:c.T3032A:p.L1011Q | Efalizumab | Biotech | DrugBank |
| PDGFRB | 5159 | 5 | 149514489 | Missense Mutation | NM_002609:exon4:c.C455T:p.T152I | Imatinib | Small molecule | FDA |
| PDGFRB | 5159 | 5 | 149515397 | Missense Mutation | NM_002609:exon3:c.A85T:p.I29F | Imatinib | Small molecule | FDA |
| RET | 5979 | 10 | 43612152 | Missense Mutation | NM_020630:exon12:c.A2257G:p.T753A | Imatinib | Small molecule | DrugBank |
| RET | 5979 | 10 | 43606856 | Missense Mutation | NM_020630:exon7:c.G1465A:p.D489N | Imatinib | Small molecule | DrugBank |
| POLG | 5428 | 15 | 89873434 | Missense Mutation | NM_001126131:exon3:c.A733G:p.I245V | Valproic acid | NA | FDA |
| POLG | 5428 | 15 | 89876827 | In Frame Deletions | NM_001126131:exon2:c.156_158del:p.52_53del | Valproic acid | NA | FDA |
| CHRNB1 | 1140 | 17 | 7359930 | Missense Mutation | NM_000747:exon11:c.T1394C:p.M465T | Galantamine | Small molecule | DrugBank |
| CHRNB1 | 1140 | 17 | 7359942 | Missense Mutation | NM_000747:exon11:c.G1406A:p.R469H | Galantamine | Small molecule | DrugBank |
| MOCOS | 55034 | 18 | 33848581 | Missense Mutation | NM_017947:exon15:c.T2600C:p.V867A | Pyridoxal phosphate | Small molecule | DrugBank |

Chr: Chromosome, MCG: My Cancer Genome, FDA: Food and Drug Administration, NA: not applicate

**Supplementary Table 10.** Prediction of cancer drug-resistance for somatic mutation genes in prostate cancer of transition zone.

| Gene Symbol | Entrez Gene Id | Chromosome | Start Position | End Position | Drug Resistance | Cancer | Variant Classification |
| --- | --- | --- | --- | --- | --- | --- | --- |
| ABCA2 | 20 | 9 | 139907219 | 139907219 | Estramustine, Mitoxantrone | Small cell lung cancer | Missense Mutation |
| MSLN | 10232 | 16 | 814058 | 814058 | Adriamycin | Non-small cell lung cancer | Missense Mutation |
| NEK2 | 4751 | 1 | 211846971 | 211846971 | Bortezomib | Multiple myeloma | Missense Mutation |
| NF1 | 4763 | 17 | 29552152 | 29552152 | Platinum-based chemotherapeutic drug | Ovarian cancer | Missense Mutation |
| AR | 367 | X | 66765174 | 66765174 | Leuprolide, Bicalutamide | Prostate cancer | In Frame Insertion |
| AR | 367 | X | 66931247 | 66931247 | Leuprolide, Bicalutamide | Prostate cancer | Missense Mutation |
| BCL2 | 596 | 18 | 60985773 | 60985773 | Cisplatin, Doxorubicin, Etoposide, | Small cell lung cancer, Non-small cell lung cancer | Missense Mutation |
| HIF1A | 3091 | 14 | 62207575 | 62207575 | Bevacizumab | Glioblastomas | Missense Mutation |
| AKT1 | 207 | 14 | 105236704 | 105236704 | Cisplatin | Small cell lung cancer | Missense Mutation |
| ITGA2 | 3673 | 5 | 52371089 | 52371089 | Gefitinib, Erlotinib | Non-small cell lung cancer | Missense Mutation |
| BRAF | 673 | 7 | 140481402 | 140481402 | Vemurafenib, Dabrafenib | Melanoma | Missense Mutation |
| ABCC2 | 1244 | 10 | 101564023 | 101564023 | Methotrexate, Etoposide, Doxorubicin, Cisplatin, Vincristine, Mitoxantrone | Non-small cell lung cancer | Missense Mutation |
| PTEN | 5728 | 10 | 89692889 | 89692889 | Trastuzumab, Platinum-based chemotherapeutic drug | Breast cancer | Missense Mutation |
| RPN2 | 6185 | 20 | 35857025 | 35857025 | Docetaxel | Breast cancer | Missense Mutation |
| ABCC4 | 10257 | 13 | 95815415 | 95815415 | Methotrexate, Thiopurines, Nucleotide analogues, Irinotecan | Neuroblastoma | Missense Mutation |
| LRP1 | 4035 | 12 | 57594931 | 57594931 | Methotrexate, Cisplatin, Doxorubicin, Ifosfamide | Osteosarcoma | Missense Mutation |
| BRCA2 | 675 | 13 | 32937526 | 32937526 | Platinum-based chemotherapeutic drug | Breast cancer, ovarian cancer | Missense Mutation |
| LRP1B | 53353 | 2 | 140990837 | 140990837 | Liposomal doxorubicin | Ovarian cancer | Missense Mutation |
| CCNA2 | 890 | 4 | 122742141 | 122742141 | Doxorubicin | Non-small cell lung cancer | Missense Mutation |
| ERBB2 | 2064 | 17 | 37864776 | 37864776 | HER2 inhibitor | Breast cancer | Missense Mutation |
| MMRN1 | 22915 | 4 | 90856783 | 90856783 | Eroposide | Small cell lung cancer | Missense Mutation |
| VEGFA | 7422 | 6 | 43748558 | 43748558 | Doxorubicin, Bevacizumab | Non-small cell lung cancer, Colorectal cancer, Glioblastoma, Renal cell carcinoma | Missense Mutation |
| MSH2 | 4436 | 2 | 47702290 | 47702290 | Methotrexate, Cisplatin, Carboplatin | Colorectal cancer, Leukemia | Missense Mutation |
| TP53 | 7157 | 17 | 7579717 | 7579717 | Etoposide, Nitrogen, Mustard, Cisplatin | Lymphoma | Missense Mutation |
